# Pathotype-specific antimicrobial resistance in diarrheagenic *Escherichia coli*: gene variants, resistance mechanisms, and evolution of treatment strategies

**DOI:** 10.3389/fmicb.2026.1770628

**Published:** 2026-03-27

**Authors:** Dhamini Kamal Raj, Sai Kiruthiga Saravanan, Anumitha Viswanathan, Santhosh Mudipalli Elavarasu, Sidharth Kumar Nanda Kumar, K. S. Sridharan, Amudha Govindarajan, Sasikumar Krishnan, Magesh Ramasamy

**Affiliations:** 1 Department of Biotechnology, Faculty of Biomedical Sciences and Technology, Sri Ramachandra Institute of Higher Education and Research (DU), Chennai, India; 2 Department of Integrative Biology, School of Biosciences and Technology, Vellore Institute of Technology (VIT), Vellore, Tamil Nadu, India; 3 Central Laboratory Services, Sri Ramachandra Medical Center, Porur, Chennai, India; 4 Department of Pharmacology, Government Medical College, Tiruvallur, India; 5 Sensor and Biomedical Technology, School of Electronics Engineering, Vellore Institute of Technology, Vellore, India

**Keywords:** antimicrobial resistance, pathotypes, phage therapy, public health, vaccine development

## Abstract

Diarrheagenic *Escherichia coli* (DEC) is still a prominent cause of diarrheal disease and mortality in children under five, and it continues to be a significant worldwide health concern. This review summarises and analyses recent findings (2015–2025) on the classification, geographic distribution, antimicrobial resistance (AMR) patterns, and emerging treatment options for six principal DEC pathotypes: enterotoxigenic (ETEC), enteropathogenic (EPEC), enteroaggregative (EAEC), Shiga toxin–producing (STEC/EHEC), enteroinvasive (EIEC), and adherent-invasive (AIEC) strains. Across diverse regions, diarrheagenic *E. coli* remains widespread in low- and middle-income populations. However, the epidemiological landscape is changing, as EAEC increasingly replaces traditional pathotypes in parts of Africa and Asia. Resistance levels to ampicillin and trimethoprim–sulfamethoxazole frequently surpass 50%, and rising resistance to fluoroquinolones, azithromycin, and third-generation cephalosporins is increasingly documented. The expansion of multidrug resistance is driven by various mechanisms, including the production of extended-spectrum *β*-lactamase (ESBL), the transfer of plasmid-borne resistance genes, the activation of efflux pumps, and the formation of biofilms. Novel interventions, including bacteriophage therapy, ETEC-focused vaccine candidates, anti-virulence agents, and approaches to modulate gut microbiota, are progressing from laboratory research to clinical evaluation. Addressing the AMR threat in DEC will need integrated One Health surveillance strategies and tailored antimicrobial stewardship measures. The current evidence highlights an urgent need for coordinated international action to reduce the clinical and public health burden associated with DEC.

## Introduction

Diarrheal diseases continue to severely affect global child health, particularly among children under five, an age group that tragically accounts for nearly half a million deaths each year (Saka et al., 2019; Acosta et al., 2016). While viral infections like rotavirus and norovirus are known drivers, bacteria such as Diarrheagenic *Escherichia coli* (DEC) are an equally pressing public health issue (Singh et al., 2019). DEC covers a range of *E. coli* types, each genetically and phenotypically adapted to cause gut infection. Over time, these bacteria have acquired specialized traits that enhance their ability to adhere to the intestinal epithelium, evade host immune defences, and produce powerful toxins (Singh et al., 2019; Akinlabi et al., 2023; Singh et al., 2019). The major DEC pathotypes are enterotoxigenic (ETEC), enteropathogenic (EPEC), enteroaggregative (EAEC), Shiga toxin–producing/enterohemorrhagic (STEC/EHEC), enteroinvasive (EIEC), and adherent-invasive (AIEC) (Table 1). Each behaves differently within the host some (such as ETEC) rely on enterotoxins, while others (such as EPEC) form attach-and-efface lesions or, in the case of STEC/EHEC, secrete powerful Shiga toxins (von Mentzer et al., 2014; Tobias et al., 2015). Globally, DEC’s burden is considerable, but not uniform. Many lower- and middle-income countries report that more than a third of paediatric diarrhea cases are due to DEC (Ren et al., 2024; Taha and Yassin, 2019; von Mentzer et al., 2014). For instance, a large-scale study in Nigeria attributed 40.4% of young children’s diarrhea episodes to DEC, mainly ETEC and EAEC (von Mentzer et al., 2014). Comparable figures come from Ethiopia, where researchers detected DEC in 38.2% of diarrheal children, with EAEC most common, followed by EPEC and ETEC (Taha and Yassin, 2019). Mongolia exhibits a similar trend, where EAEC and EPEC are the predominant isolates (Boisen et al., 2020; Taha and Yassin, 2019). Clearly, DEC is not limited to resource-limited settings; evidence also links it to severe pediatric cases in high-income countries such as Israel (Bryan et al., 2015). Interestingly, the distribution of DEC types varies. Meta-analyses focusing on Asia list ETEC leads with (49%), followed by EPEC (31%), EAEC (13%), STEC (5%), and EIEC (2%) (Boisen et al., 2020; Pasqua et al., 2017; Ren et al., 2024). According to recent research, EAEC is already posing a threat to ETEC’s hegemony in a number of Asian and African regions between 2022 and 2025 (Boisen et al., 2020; Pasqua et al., 2017). Scientists speculate that this alteration may be related to changes in the environment, easy access to potable water, or even more animal contact (Perna et al., 2020; Gilligan, 1999). The identification of mixed infections involving several DEC subtypes is growing, which complicates diagnosis and treatment (Boisen et al., 2020; Gilligan, 1999; Bryan et al., 2015).

**Table 1:** *Escherichia coli* pathotypes with associated diarrheal types and clinical symptoms.

Pathotypes	Diarrheal type	Clinical symptoms
Enterotoxigenic *Escherichia coli* (ETEC)	Traveller’s diarrhoea	Watery, non-bloody diarrhoea and vomiting
Enteropathogenic *Escherichia coli* (EPEC)	Infant diarrhea	Persistent watery diarrhoea and vomiting
Enteroaggregative *Escherichia coli* (EAEC)	Traveller’s diarrhoea and infant diarrhoea	Persistent watery diarrhoea with mucus
Shiga toxin–producing *Escherichia coli* (STEC)	Haemorrhagic colitis,Haemolytic uremic disease	Bloody diarrhea
*Enteroinvasive Escherichia coli* (EIEC)	Dysentery	Bloody diarrhoea with mucus
Adherent-invasive *Escherichia coli* (AIEC)	Associated with Crohn’s disease	Persistent intestinal inflammatory diarrhoea

Source: Nataro and Kaper (1998), Qadri et al. (2005), Lan et al. (2001), Tarr et al. (2005).

When antimicrobial resistance (AMR) is involved, the problem becomes more difficult. Although many DEC infections heal without the use of antibiotics, severe instances do necessitate medication, particularly in young children or those with weakened immune systems (Khalil et al., 2021). Multidrug resistance (MDR) has been reported at alarming rates: in Asia, about half of the analyzed DEC isolates produce extended-spectrum *β*-lactamase enzymes, and approximately two-thirds are MDR (Khalil et al., 2021; Pasqua et al., 2017). The rates are startlingly similar elsewhere: investigations conducted in Mongolia revealed that 68% of pediatric DEC isolates were resistant to TMP-SMX and β-lactams, while over 43% of bacteria in Ethiopia were MDR and some even produced carbapenemases (Taha and Yassin, 2019). In peri-urban regions of Kenya, the issue affects both children and livestock, studies show that over 60% of DEC isolates show multidrug resistance (MDR) (Gilligan, 1999; Taha and Yassin, 2019). Beyond just failing to respond to treatment, these resistant strains can survive for long periods of time in both human and animal hosts as well as the environment, and they may spread more easily than their non-resistant counterparts (Karami et al., 2024). Antimicrobial-resistant DEC strains may spread more efficiently than their non-resistant counterparts because resistance genes are frequently carried on mobile genetic elements such as plasmids, transposons, and integrons, which often also harbor virulence factors. This genetic linkage allows resistant strains to survive antibiotic pressure in both clinical and community settings, leading to prolonged colonization and higher chances of transmission. In addition, antibiotic exposure can eliminate susceptible competing bacteria in the gut microbiota, giving resistant DEC strains a selective advantage and facilitating their persistence and spread (Karami et al., 2024). Furthermore, urbanization, globalization, and climate change are all contributing to the spread of DEC and associated resistance genes by increasing population density and sanitation pressures in rapidly expanding urban settings, enhancing bacterial survival and persistence under rising temperatures, facilitating cross-border transmission through international food trade, travel and intensifying interactions between humans, livestock and environmental reservoirs that promote horizontal transfer of mobile resistance elements (Xiong et al., 2024). The global effect, the ever-changing epidemiology, and the threat posed by AMR make DEC a legitimate top priority for diarrhoea control globally. There is an urgent need for updated analysis due to the prevalence of DEC, new trends among its many pathotypes, and the emergence of antibiotic resistance all of which have significant implications for surveillance, diagnosis, development of novel treatment and preventive techniques.

### Classification and prevalence of DEC pathotypes

**Figure 1:**
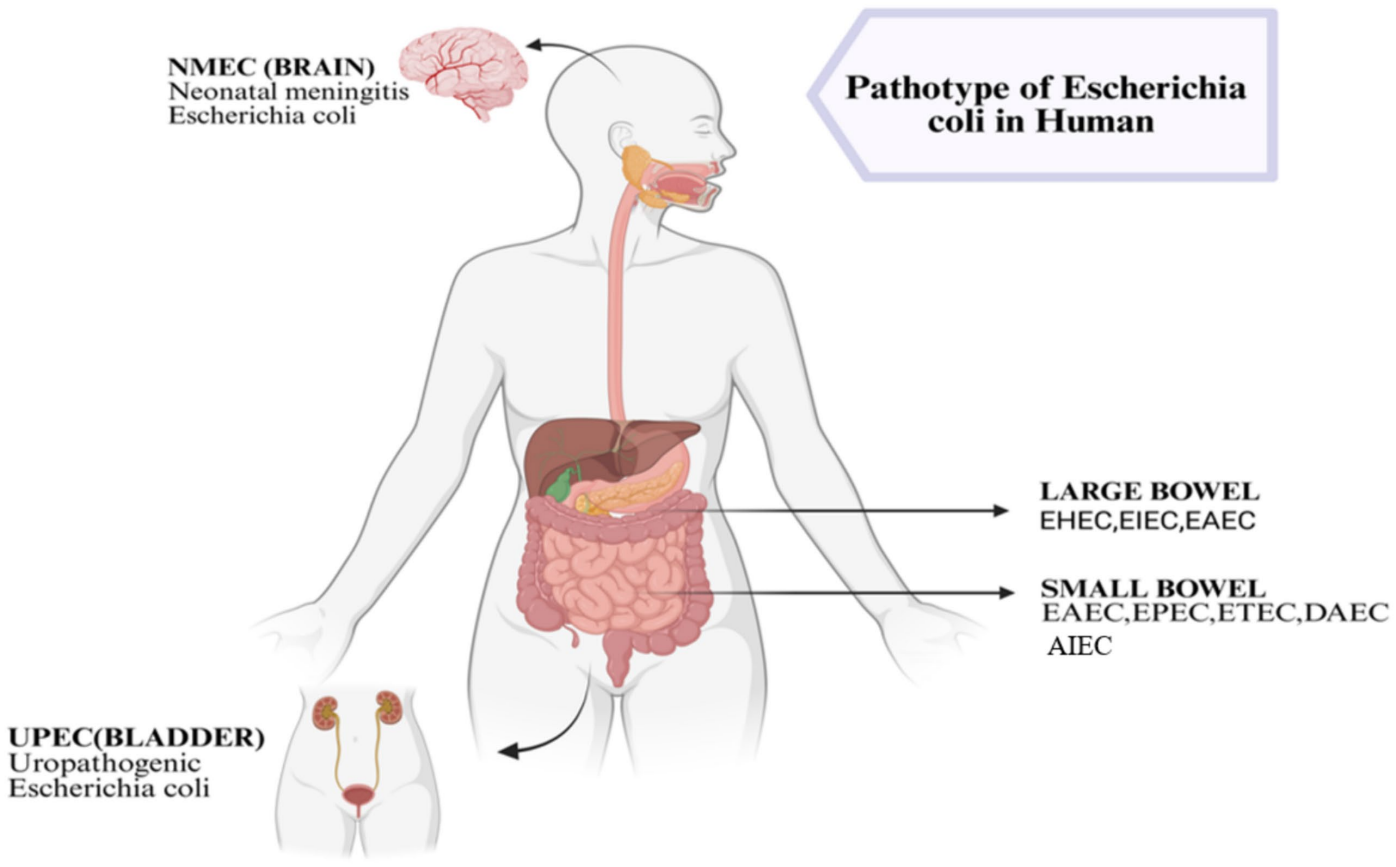
The major pathotypes of *Escherichia coli* in the human body, highlighting their primary sites of infection. The schematic illustrates NMEC targeting the brain, causing neonatal meningitis, UPEC affecting the bladder and urinary tract, and enteric pathotypes colonising either the large or small intestine. Specifically, the large bowel is targeted by EHEC, EIEC, and EAEC, while the small bowel is affected by EAEC, EPEC, ETEC, and DAEC.

*Escherichia coli* strains classified as “diarrheagenic” (DEC) possess distinct virulence factors, pathogenic processes, and ecological niches. Globally, diarrhea is still mostly caused by these numerous pathotypes. Children are shown to be disproportionately affected in low- and middle-income countries (LMICs). Their significance keeps growing as food supply chains shift, molecular diagnostics become more widely available, and antibiotic resistance affects infection dynamics. This section describes the main types of DECs, summarizes global distribution trends, and provides recent prevalence data from 2022 to 2025. The major diarrheagenic *Escherichia coli* pathotypes differ in their virulence characteristics, intestinal localisation and associated clinical manifestations as illustrated in (Figure 1).

ETEC producing heat-labile (LT) and/or heat-stable (ST) toxins, remains a leading cause of childhood and travellers’ diarrhoea, accounting for approximately 10–20% of paediatric diarrhoeal cases in endemic regions (Mandal and Sahi, 2017; EFSA BIOHAZ Panel et al., 2020). EPEC characterised by attaching-and-effacing lesions mediated by the LEE pathogenicity island, contributes an estimated 5–15% of childhood diarrhoea cases (Sukwa et al., 2023; Song et al., 2023). EAEC known for its aggregative adherence and biofilm formation, is increasingly associated with acute and persistent diarrhoea, with reported prevalence ranging from 5–20% (Elst, 2024; Gaastra and Svennerholm, 1996). STEC/EHEC responsible for bloody diarrhoea and haemolytic-uraemic syndrome, accounts for approximately 1–5% of global cases, including both O157 and non-O157 serogroups (Liu et al., 2022; Ren et al., 2024). EIEC is less frequently detected (<3%) but implicated in dysentery-like illness and occasional outbreaks (Heredia and García, 2018; Pasqua et al., 2017). AIEC is primarily linked to inflammatory bowel disease, particularly Crohn’s disease, with colonisation rates reported at 20–40% among affected patients (Martinez-Medina et al., 2009; Perna et al., 2020).

## ETEC

ETEC-The heat-labile (LT) and/or heat-stable (ST) toxins that cause ETEC infections are characterized by watery stools (Table 1). These stimulate secretory diarrhoea without penetrating deeply into intestinal tissues. The organisms secure themselves to enterocytes using colonisation factors (CFs), allowing direct toxin delivery. In clinical terms, ETEC is a major cause of infantile diarrhoea in endemic regions and is also the best-known cause behind travellers’ diarrhoea (Mandal and Sahi, 2017; EFSA BIOHAZ Panel et al., 2020; Qadri et al., 2005).

### Pathogenesis and virulence mechanism

**Figure 2:**
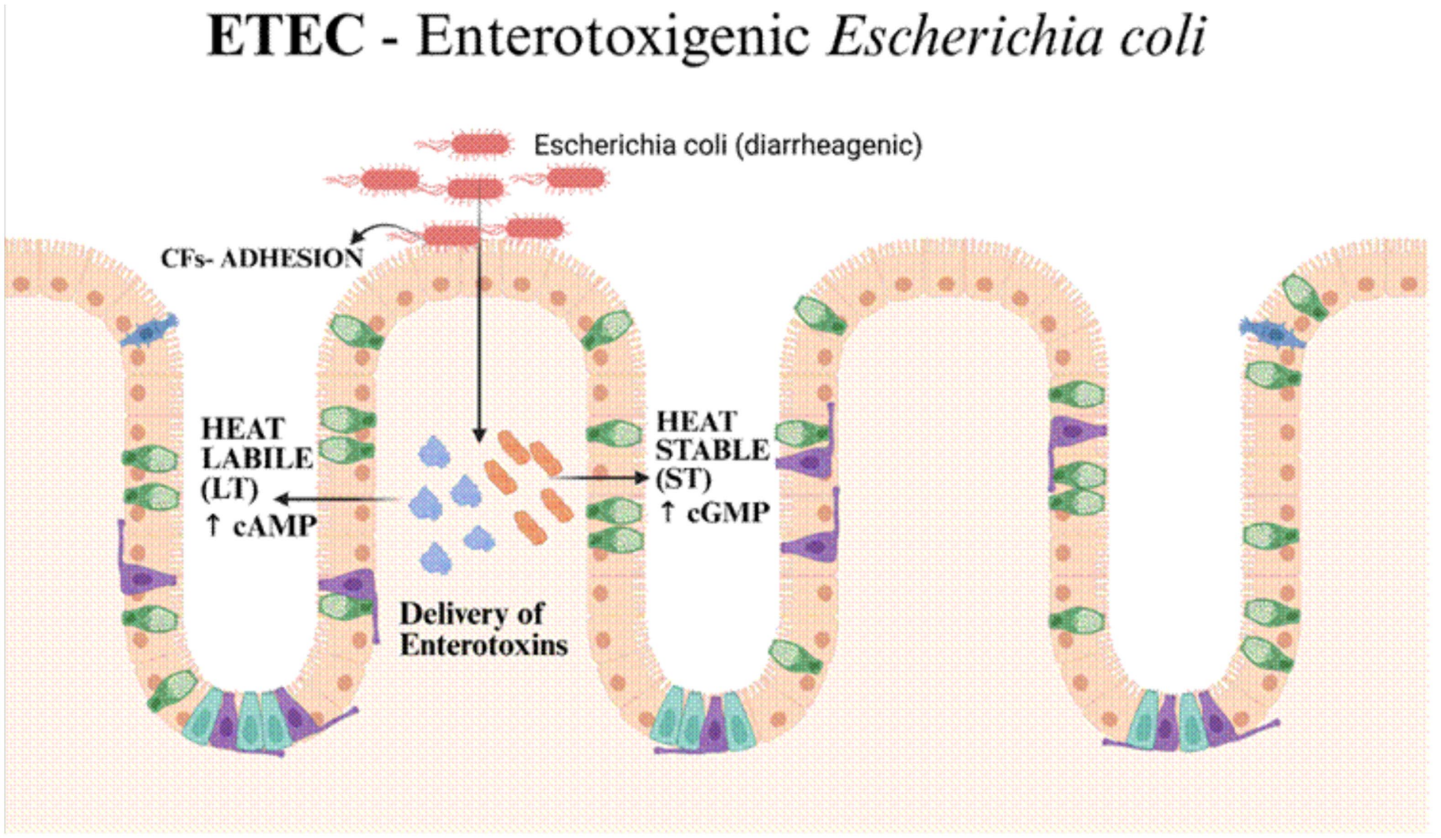
ETEC first attaches to the lining of the small intestine using specialised colonisation factors (CFs) that help it adhere firmly to the mucosal surface. Once connected, the bacteria release heat-labile (LT) and heat-stable (ST) toxins into the intestinal cells. These toxins activate cAMP- and cGMP-dependent signalling pathways, disrupting ion movement and fluid balance in the gut, which ultimately leads to secretory diarrhoea.

The pathogenicity of this strain is primarily due to two plasmid-encoded enterotoxins, heat-labile (LT) and heat-stable (ST), that act synergistically (Figure 2). These toxins cause severe, watery diarrhoea without actually invading the gut lining (Croxen and Finlay, 2010). Similar to the cholera toxin, LT is an AB5 toxin. It attaches itself to a particular chemical known as GM1 gangliosides found on intestinal cells. This binding initiates a series of events that increase cAMP levels by activating a protein known as adenylate cyclase. When CFTR chloride channels are opened by this spike in cAMP, a massive volume of fluid leaks into the stomach (Croxen and Finlay, 2010). ST toxins are different; they are tiny peptides and they activate a different protein, guanylate cyclase C, which raises cGMP inside the cells. This causes a surge of bicarbonate and chloride to exit the cells while also preventing the absorption of sodium. As a consequence, the watery stools that are indicative of an ETEC infection development (Croxen and Finlay, 2010; Qadri et al., 2005).

### Colonisation factors and intestinal attachment

ETEC uses a whole bunch of colonisation factors (CFs) (Figure 2), which are either fimbrial or non-fimbrial. The classic ones like CFA/I and CS1–CS7, but there are tons of other versions around the world, often specific to certain places. These CFs, which are also encoded on plasmids, serve as anchors, securing the ETEC bacteria to the intestinal lining and facilitating the formation of mini-colonies, allowing them to distribute their toxins precisely where they are needed. The fact that these CFs can mix and match with different toxins makes it really tough to keep an eye on the bug and to create effective vaccines (Gaastra and Svennerholm, 1996; Natarajan et al., 2018).

### Clinical features and global epidemiology

There is usually no blood or significant inflammation, but symptoms include dehydration, cramping in the stomach and abrupt watery diarrhoea (Qadri et al., 2005; Croxen and Finlay, 2010) (Table 1). In one study following a group of kids in Peru from birth, almost seven out of ten had at least one ETEC episode by the time they were two years old. Its early impact on a child’s health was demonstrated by the rate, which was 73 instances per 100 child-years (Platts-Mills et al., 2015). In Bangladesh, hospital data showed a jump in ETEC from 7.4% in 2017 to 10.5% in 2022, which suggests that ETEC remains a significant enteric pathogen, with improved diagnostic capacity potentially contributing to higher reported prevalence (Qadri et al., 2005). ETEC is still a major problem, even if the prevalence of diarrhoea varies by region, according to a study conducted in Zambia, a country in sub-Saharan Africa, which found 2.47 episodes per 100 child-years (Simuyandi et al., 2019). More recently, a hospital study in a cholera-prone area of India found ETEC in over 20% of diarrhoea admissions, making it the most common cause in that specific setting. All these studies confirm that ETEC is a persistent threat on every continent, with a particularly heavy impact on young children.

### Diagnosis and antimicrobial resistance

Diagnosing ETEC is not as simple as a regular culture because it looks just like the harmless *E. coli* that lives in our guts. Instead, laboratories must detect the specific *elt* (LT) and *est* (ST) toxin genes using molecular methods such as PCR, and in some cases, they also test for genes encoding colonization factor (CF) (Qadri et al., 2005). Advanced techniques like multiplex PCR and ELISA for LT/ST detection have enhanced diagnostic accuracy, their use remains limited in resource constrained settings (Isidean et al., 2011). Rehydrating the patient is the mainstay of treatment, while severe cases may occasionally require the use of antibiotics. The main challenge is rising multidrug resistance (MDR). A large study carried out across Asia found that many ETEC strains produce ESBL enzymes, and quite often, the genes responsible for both toxin production and antibiotic resistance are carried on the same plasmids mobile pieces of DNA that can easily move between bacteria (Salleh et al., 2022). This explains why we are seeing more and more ETEC strains that are not highly infectious but also difficult to treat (Nasrollahian et al., 2024).

### Prevention

The main strategy for preventing ETEC infection is vaccine development. Some oral vaccines that include different CFs have shown partial protection, and LT-based toxoid vaccines have shown a strong immune response in clinical trials, but the protection they offer has been limited so far (Gaastra and Svennerholm, 1996; Nasrollahian et al., 2024). Interestingly, infants, receive early immune protection from their mothers’ antibodies, which highlights the importance of breastfeeding is so important in these areas (Croxen and Finlay, 2010). In recent years, scientists have begun turning their attention to other parts of the bacterium, such as outer-membrane antigens, and are applying a method known as reverse vaccinology to overcome the difficulties caused by the wide range of colonization factors (CFs). Even with these advances, a broadly protective and approved vaccine is still not available. Until that changes, preventing ETEC mainly depends on improving sanitation, providing access to clean water, and maintaining proper hygiene. Responsible use of antibiotics is equally important to slow the rise of antimicrobial resistance (Salleh et al., 2022).

## EPEC

The distinguishing feature of EPEC infection is the creation of attaching-and-effacing (A/E) lesions, a process orchestrated by genes located on the Locus of Enterocyte Effacement (LEE) pathogenicity island. Critical to its disease-causing ability are the adhesion molecule intimin (*eae*) and a type III secretion system. Two variants are recognised: typical EPEC (tEPEC), which contains a plasmid encoding bundle-forming pili (*bfpA*), and atypical EPEC (aEPEC), which lacks this plasmid but remains pathogenic (Sukwa et al., 2023; Song et al., 2023; Sukwa et al., 2023).

### Prevalence and public health importance

EPEC remains a frequent finding in research on childhood diarrhoea. For example, a 2015 study conducted in Baghdad detected the pathogen in roughly one-third of all laboratory samples, underscoring its widespread presence. EPEC is not only being detected in patients with diarrheal illness; food safety investigations have also identified it in animal-derived foods, indicating that the organism may be circulating through the food chain (Taha and Yassin, 2019). In India, numerous hospital-based studies have likewise documented considerable numbers of EPEC cases, though the reported prevalence varies by region and depending on whether atypical strains were included in the assessment (Singh et al., 2019). EPEC was one of the first *E. coli* types ever identified as a cause of diarrhoea and remains a leading cause of severe diarrhoea in infants in developing nations (Levine, 1987).

### Attaching and effacing lesion formation and virulence machinery

**Figure 3:**
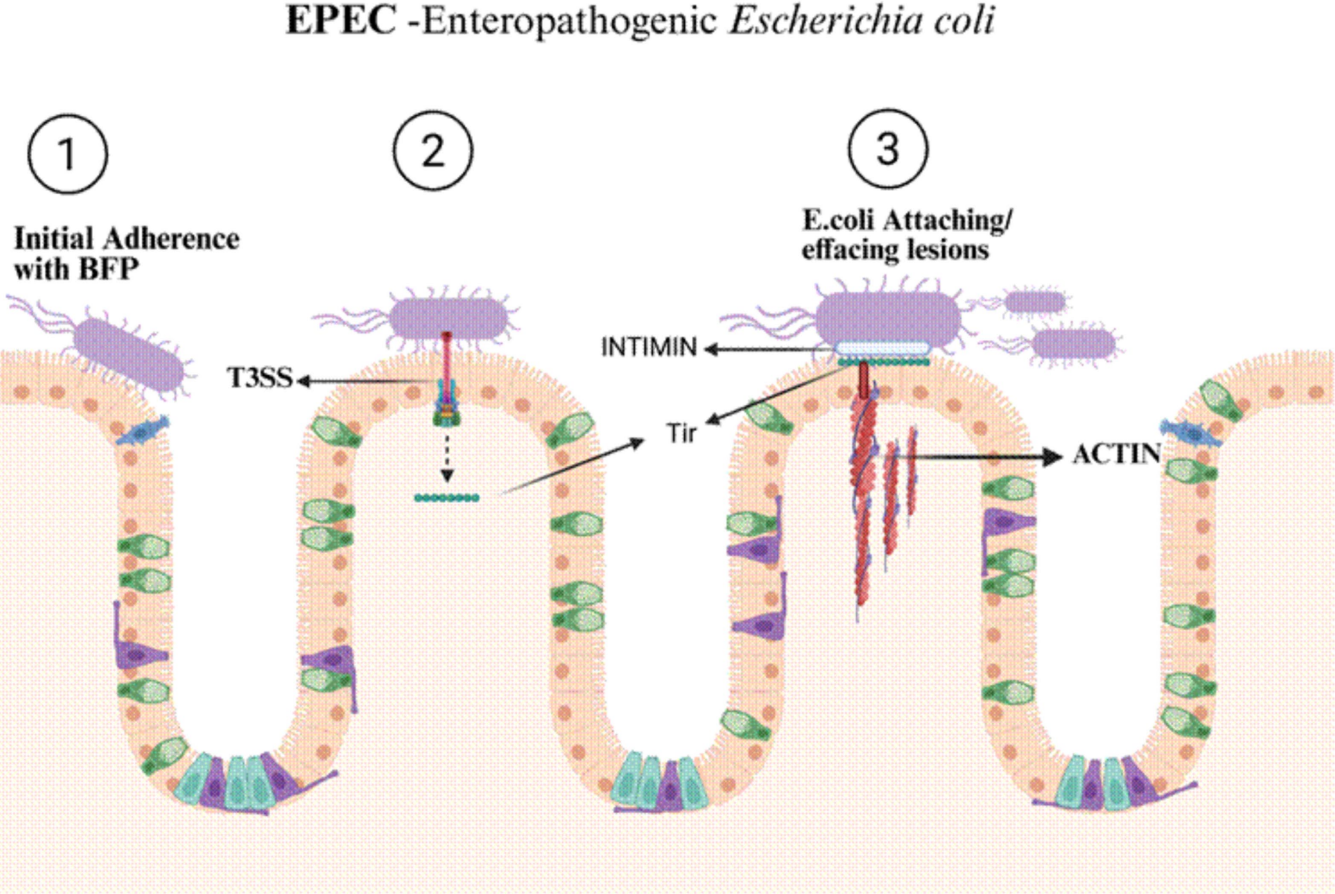
EPEC initiates infection through localised adherence to the intestinal epithelium via bundle-forming pili (BFP), followed by delivery of effector proteins through a type III secretion system. These effectors induce attaching-and-effacing (A/E) lesions, characterised by microvilli effacement and actin pedestal formation, resulting in the disruption of absorptive surface integrity and malabsorptive diarrhoea.

Its key feature is its ability to create attaching-and-effacing (A/E) lesions (Table 2) on the lining of the small intestine (Figure 3). The virulence of this pathotype is primarily driven by a large chromosomal pathogenicity island known as the locus of enterocyte effacement (LEE). The LEE acts like a tiny syringe, a type III secretion system (T3SS), to inject proteins into gut cells, including the adhesin intimin (Croxen and Finlay, 2010). After the EPEC has initially settled in, it injects a protein called Tir into the gut cells (Figure 3). Tir then inserts itself into intestinal epithelial cell membrane of the host, acting as a receptor for intimin on the surface of EPEC and allowing the bacterium to attach very tightly to the cell. This close interaction triggers a rearrangement of the cell’s internal framework, leading to the formation of pedestal-like structures and the destruction of the tiny, finger-like microvilli (Croxen and Finlay, 2010; Wong et al., 2011). Other injected proteins, such as EspF, Map, and EspG, enhance fluid loss and diarrhoea by interfering with cell energy, rupturing cell-to-cell barriers, and changing ion channel function (Wong et al., 2011).

**Table 2:** Antimicrobial resistance genes, virulence factors, and resistance mechanisms among diarrheagenic *Escherichia coli* pathotypes.

Pathotypes	Antimicrobial resistance gene	Virulence factor	Antimicrobial resistance mechanism
ETEC	*blaTEM, tetA, sul1, dfrA, aadA*	Colonisation factors, heat-labile and heat-stable toxins	β-lactamase production (e.g., *blaTEM*); Efflux pumps; Porin channel mutations; Plasmid-mediated resistance
EPEC	*blaTEM, catA, sul2, tetB, qnrS*	Adhesins: Bundle-forming pili, intimin and locus of enterocyte effacement	β-lactamase production; Efflux pumps; Plasmid-mediated resistance
EAEC	*blaCTX-M, tetA, sul1, aadA, qnrB*	Adhesins: Aggregative adherence fimbriae (AAF), enterotoxins, Biofilm formation	Extended-spectrum β-lactamases (e.g., *blaCTX-M*); Efflux pumps; Plasmid-mediated resistance
STEC	*blaTEM, qnrA, tetA, sul2, aadA*	Shiga toxin: Stx1 and Stx2, Adhesins, Intimin, locus of enterocyte effacement (LEE)	β-lactamase production; Efflux pumps; Plasmid-mediated resistance
EIEC	*blaTEM, tetA, sul1, catA, dfrA*	Cellular Invasions, Ipa proteins, actin-based motility, hemolysin	β-lactamase production; Efflux pumps; Plasmid-mediated resistance
AIEC	*blaCTX-M, blaTEM, tetA, sul1, qnrB, aac(3)-IIa*	Type 1 fimbriae, Cellular invasion, intracellular survival, adhesins	Extended-spectrum β-lactamases (e.g., *blaCTX-M*); Efflux pumps; Plasmid-mediated resistance

Source: Nasrollahian et al. (2024).

### Typical and atypical strains with evolving transmission patterns

There are two major types of EPEC strains: typical (tEPEC) and atypical (aEPEC). The gene for bundle-forming pili (BFP) is found on the EAF plasmid, an additional plasmid found in typical EPEC. The bacteria adhere to one another and create mini-colonies with the aid of these pili. Although atypical EPEC does not have this plasmid, it is nonetheless harmful since it contains the LEE pathogenicity island and other means of adhering to cells (Trabulsi et al., 2002). Historically, tEPEC was the main cause for infant diarrhoea in places like Latin America, Africa, and Asia but studies are now showing that aEPEC is the more common type worldwide, causing both isolated cases and outbreaks (Trabulsi et al., 2002). Atypical EPEC strains have shown extraordinary adaptability, with hybrid versions which share virulence factors with other types of *E. coli*, that makes them even harder to classify and raises concerns about its potential to cause even more severe disease (Hernandes et al., 2009). The fact that EPEC is found in animals and in foods like milk, beef, and fresh produce points to its ability to spread from animals to humans, adding a food-borne transmission route on top of direct person-to-person spread (Taha and Yassin, 2019).

### Clinical manifestation and immune evasion

Clinically, an EPEC infection is known for causing watery diarrhoea (Table 1) that can be prolonged and severe, especially in children under two, and it’s often accompanied by fever, vomiting, and dehydration. Unlike some other types of *E. coli*, EPEC does not typically cause bloody diarrhoea. However, its tendency to cause persistent diarrhoea significantly contributes to malnutrition and stunted development in regions where it’s common (Levine, 1987). Studies that have followed children from birth in Latin America and Asia have confirmed that EPEC is one of the top causes of diarrhoea in infants. Meanwhile, hospital surveillance in India and Africa shows that both typical and atypical strains are circulating at high rates, with aEPEC now making up the majority of isolates in many regions (Mwape et al., 2023). EPEC has developed multiple strategies to evade host immune defences by injecting effector proteins such as Nle family members that suppress inflammatory signalling pathways, disrupt epithelial barrier integrity, and interfere with host cell recruitment and cytokine responses, allowing the bacteria to persist longer within the intestinal mucosa, particularly in young children whose immune systems are still developing (Wong et al., 2011). Others mess with the balance of cell death and survival, weakening the body’s defences. While the host’s immune system does try to fight back by sending in immune cells and releasing inflammatory proteins, it’s often not enough to clear the infection quickly, which is why it can persist in young children (Croxen and Finlay, 2010).

### Diagnosis, antimicrobial resistance, and vaccine challenges

EPEC cannot be distinguished from non-pathogenic intestinal *E. coli* strains that form part of the normal gut microbiota. This is because typical culture and biochemical identification methods detect *E.coli* at the species level but do not differentiate pathogenic strains from non-pathogenic commensals, as both share similar morphological and metabolic characteristics; therefore, molecular detection of virulence genes such as eae and bfpA is required for accurate identification of EPEC. Therefore, to validate the presence of EPEC by identifying the *eae* (encodes intimin) gene, PCR is required. Additionally, screening for the bfpA gene can also assist with the differentiation of typical and atypical EPEC (Trabulsi et al., 2002). However, through advanced methods of analysis such as multiplex PCR and next generation sequencing, hybrid strains have been identified carrying EPEC genes along with genes from other strains of *E. coli*; this demonstrates the ability of these bacteria to evolve and continue to develop (Hernandes et al., 2009). While oral or Intravenous rehydration represents the primary aspect of the generally supportive nature of treatment, due to the increasingly high levels of antibiotic resistant EPEC strains, the use of antibiotics to treat EPEC infections has become less effective; particularly when used to treat more serious or chronic infections. High levels of antibiotic resistance have recently been reported in India and Africa, specifically against common antibiotics including fluoroquinolones, cephalosporins and aminoglycosides; this raises concerns regarding the use of antibiotics to effectively treat EPEC infections in high burden areas (Natarajan et al., 2018).

In light of the potential danger associated with EPEC infection combined with the difficulty in treating the infection with antibiotics, EPEC presents a unique two-pronged problem to both the control of infectious diseases and the prudent use of antibiotics. As a vaccine target, EPEC has proven to be a difficult target to create due to the diversity of the surface proteins present on the bacterium and the multiple mechanisms by which it produces disease. In contrast to ETEC for which toxoid based vaccines are currently in clinical trial development, there is currently no licensed vaccine for EPEC. Experimental vaccines have included ones based on the intimin protein and weakened live strains, but the protection they offer has been limited and incomplete (Levine, 1987; Trabulsi et al., 2002).

Current research is looking into more stable parts of the bacteria, like outer-membrane proteins and components of the T3SS, as possible targets for a universal vaccine (Hernandes et al., 2009). In conclusion, EPEC remains a globally significant pathogen whose behaviour is changing, with atypical strains now dominating. Its ability to create those characteristic A/E lesions, evade the immune system, and persist in our food, combined with the growing problem of multidrug resistance, makes it clear that we urgently need to keep a close eye on it, improve our diagnostics, and develop new vaccine strategies to control its impact on child health.

## EAEC

EAEC cells display a distinctive aggregative adherence pattern, often described as resembling stacked bricks. They readily form biofilms and harbour a diverse array of virulence factors, including aggregative adherence fimbriae and toxins such as Pet and EAST-1. EAEC is linked to both short-lived and chronic diarrhoea, and recurrent infection has been associated with growth and developmental problems in children (Elst, 2024; Gaastra and Svennerholm, 1996).

### Prevalence and public health significance

Several recent studies place EAEC among the top DEC types recovered from both community and hospital cases. For example, a rural Peruvian paediatric cohort ranked EAEC as one of the most frequent isolates (Acosta et al., 2016), while in an Indian clinical series the prevalence reached about 37%. Given the established association of EAEC, particularly *aggR*-positive strains, with prolonged diarrhoea and nutritional deficits in children, its consistent appearance in these settings is a major public health concern (Singh et al., 2019; Acosta et al., 2016).

### Aggregative adherence pattern and virulence architecture

**Figure 4:**
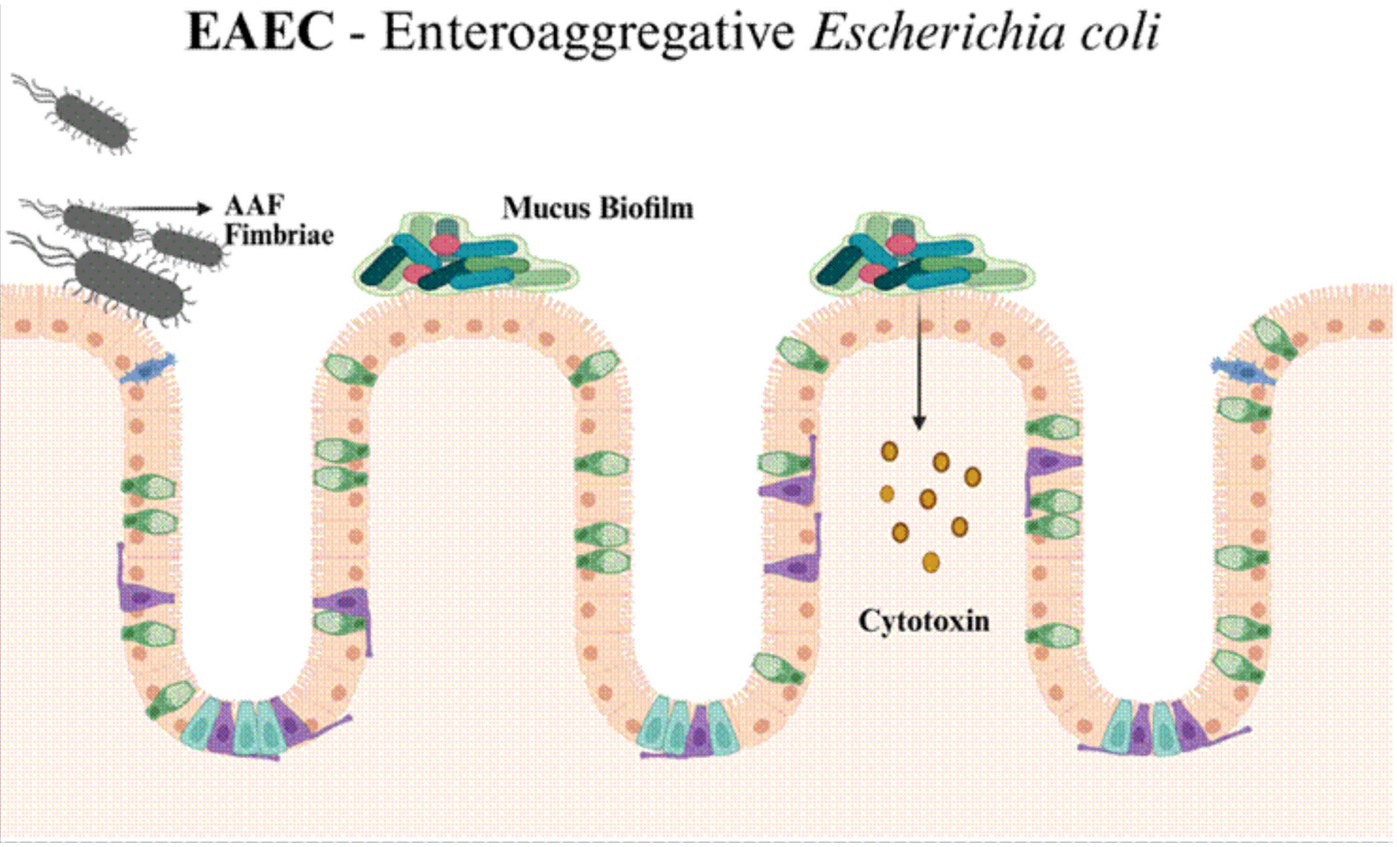
EAEC exhibits a characteristic “stacked-brick” aggregative adherence pattern on the intestinal surface via aggregative adherence fimbriae (AAF). The biofilm-like mucosal colonisation is followed by the release of enteroaggregative toxins and proinflammatory mediators, which disrupt epithelial barrier integrity and promote persistent, low-grade intestinal inflammation, leading to chronic or recurrent diarrhoea.

EAEC is defined phenotypically by its characteristic “stacked-brick” aggregative adherence (Table 2) to HEp-2 or HeLa cells, a pattern driven primarily by aggregative adherence fimbriae (AAF) (Figure 4) variants that mediate tight interbacterial and bacteria–epithelial interactions and promote biofilm formation on the intestinal mucosa (Nataro and Kaper, 1998). Central to EAEC pathogenicity is a modular virulence architecture often encoded on plasmids and chromosomes that includes the transcriptional regulator AggR (a master activator of an EAEC virulence regulon), AAF adhesins (multiple allelic variants), dispersin and its secretion system (aat), autotransporters such as Pic and the serine protease Pet (a SPATE family protease), and enterotoxins such as EAST-1; the combination and presence/absence of these factors varies widely between strains, producing a spectrum of pathogenic potential from asymptomatic colonisation to persistent, inflammatory diarrhoea (Nataro and Kaper, 1998; Sheikh et al., 2001).

### Biofilm formation and epithelial damage

EAEC’s propensity to form thick, mucosa-associated biofilms is a central pathogenic trait: biofilm matrices composed of fimbriae, secreted proteins and extracellular polymeric substances enable prolonged mucosal colonisation, resistance to flushing, and protection from host innate defences and some antimicrobials,thus leading to persistent diarrhoeal symptoms and recurrent infections noted in epidemiological investigations (Nataro and Kaper, 1998; Sheikh et al., 2001; Harrington et al., 2006). Toxins and autotransporters contribute to mucosal damage and fluid loss: Pet can cleave cytoskeletal and junctional proteins leading to cell rounding and detachment, while Pic has mucinolytic activity and can modulate immune responses; EAST-1 and other small toxins perturb ion transport and contribute to secretory diarrhoea in susceptible hosts (Harrington et al., 2006; Sheikh et al., 2001). The net effect intimate adherence, biofilm persistence, toxin-mediated epithelial disruption, and modulation of immune signalling explains why EAEC can cause both acute and chronic diarrhoea and why it is particularly detrimental to child growth and nutrition.

### Epidemiology and chronic disease outcomes

Epidemiologically, EAEC behaves as both an endemic pathogen and an agent of outbreaks; it is frequently recovered in community-based surveillance (including the Peruvian cohort noted above) and in hospital panels from LMICs, and has been implicated in travel-associated diarrhoea as well (Acosta et al., 2016; Liu et al., 2022). Heterogeneity in detection rates between studies reflects differences in diagnostic methods (culture vs. PCR vs. HEp-2 adherence assays), patient selection (community cohorts vs. hospitalised patients), and the molecular criteria used to define EAEC (presence of virulence genes such as *aggR, aaf/aggA, aatA*, etc.), which together can expand or narrow the case definition and thus reported prevalence (Aslani et al., 2011; Nataro and Kaper, 1998). Several prospective pediatric studies have linked EAEC infection with prolonged episodes, higher rates of stool pathogen persistence, and measurable impacts on growth parameters (weight-for-age, height-for-age), suggesting that repeated or chronic EAEC exposure contributes to malnutrition and developmental consequences in endemic settings (Acosta et al., 2016; Liu et al., 2022; Rogawski et al., 2017).

### Diagnosis and molecular detection

Laboratory diagnosis has moved towards molecular markers because the classical HEp-2 adherence assay is laborious and not routinely feasible in many settings; common PCR targets include *aggR* (the transcriptional activator associated with typical EAEC strains), *aatA* (the dispersin secretion gene), *aaf/aggA* variants for fimbrial types, and pet or pic when searching for toxin/autotransporter presence (Nataro and Kaper, 1998; Aslani et al., 2011). Nevertheless, no single molecular marker perfectly predicts pathogenicity across all EAEC isolates some *aggR*-negative strains can be pathogenic and some *aggR*-positive isolates can be carried asymptomatically so a combination of molecular markers plus clinical correlation tends to give the most accurate case definition (Nataro and Kaper, 1998; Aslani et al., 2011). Whole-genome sequencing in recent studies has further clarified the EAEC pan-genome and highlighted frequent recombination and co-occurrence of plasmid-borne virulence cassettes, explaining both the stability of certain clones and the rapid emergence of new, locally dominant lineages (Hernandes et al., 2009; Nataro and Kaper, 1998).

### Treatment, resistance trends, and prevention

Clinically, management of EAEC focuses on rehydration and nutritional rehabilitation; antibiotics are generally reserved for severe disease or for outbreak control and should be guided by susceptibility testing because EAEC frequently carries mobile resistance determinants (Rogawski et al., 2017). Indeed, surveillance and meta-analyses within the region show high rates of resistance among EAEC isolates to commonly used oral antibiotics (e.g., ampicillin, trimethoprim-sulfamethoxazole) and emerging resistance to fluoroquinolones and third-generation cephalosporins via ESBLs in some locales, complicating empirical therapy and increasing the need for local susceptibility data to drive treatment algorithms (Nasrollahian et al., 2024; Salleh et al., 2022).

The biofilm lifestyle of EAEC can also reduce antibiotic penetration and efficacy, meaning that even phenotypically susceptible isolates may be difficult to eradicate from the gut mucosa *in vivo* (Harrington et al., 2006; Salleh et al., 2022). Beyond acute therapy, EAEC control requires improved diagnostics, water/sanitation/hygiene interventions, and nutritional programs to mitigate the long-term sequelae of chronic or recurrent infections (Acosta et al., 2016; Liu et al., 2022; Rogawski et al., 2017). Vaccine development for EAEC has been limited by the pathogen’s heterogeneity; however, candidate strategies focus on conserved adhesins, anti-biofilm targets, and toxin neutralisation, with the understanding that a broadly protective EAEC vaccine will likely need to address multiple virulence modules or target highly conserved functions critical for mucosal persistence (Nataro and Kaper, 1998; Rogawski et al., 2017; Sheikh et al., 2001).

Finally, the public-health importance of EAEC is magnified by its capacity to act as a reservoir of mobile virulence and resistance elements that can be transferred to other *E. coli* lineages, contributing to the emergence of hybrid or more virulent strains an evolutionary dynamic documented in genomic studies of diarrhoeagenic *E. coli* (Hernandes et al., 2009; Nataro and Kaper, 1998; Nasrollahian et al., 2024).

## STEC/EHEC

EHEC/STEC–hese strains produce Shiga toxins (Stx1 and/or Stx2) capable of triggering bloody diarrhoea and, in some patients, the potentially fatal haemolytic-uraemic syndrome (HUS). While the O157: H7 serotype is historically most prominent, there is increasing recognition of non-O157 serogroups as important causes of human illness (Liu et al., 2022; Ren et al., 2024).

### Serotypes, reservoirs, and food-borne transmission

STEC comprise a heterogeneous group of serotypes defined by their ability to produce Shiga toxins (Stx1 and/or Stx2). Among these, O157: H7 remains the most recognised serotype in high-income countries, recent studies show that non-O157 serotypes including O26, O45, O103, O111, O121, and O145 are also globally important. These are common in LMICs and frequently isolated from cattle and food products (Croxen and Finlay, 2010; Tarr et al., 2005; Brooks et al., 2005). Asymptomatic STEC carriage in cattle plays a major role in transmission. In most LMICs, STEC tends to be detected more often in studies of the food chain than in routine checks for paediatric diarrhoea. For instance, a large-scale investigation in China that sampled meat in 2021 found STEC present in about 4.1% of raw products, together with ETEC and EPEC (Ren et al., 2024). Broader farm and produce surveillance has also revealed strains carrying the *stx* gene, often belonging to non-O157 types, which highlights an ongoing risk of zoonotic transmission (Heredia and García, 2018; Liu et al., 2022).

### Shiga toxin biology and virulence mechanisms

**Figure 5:**
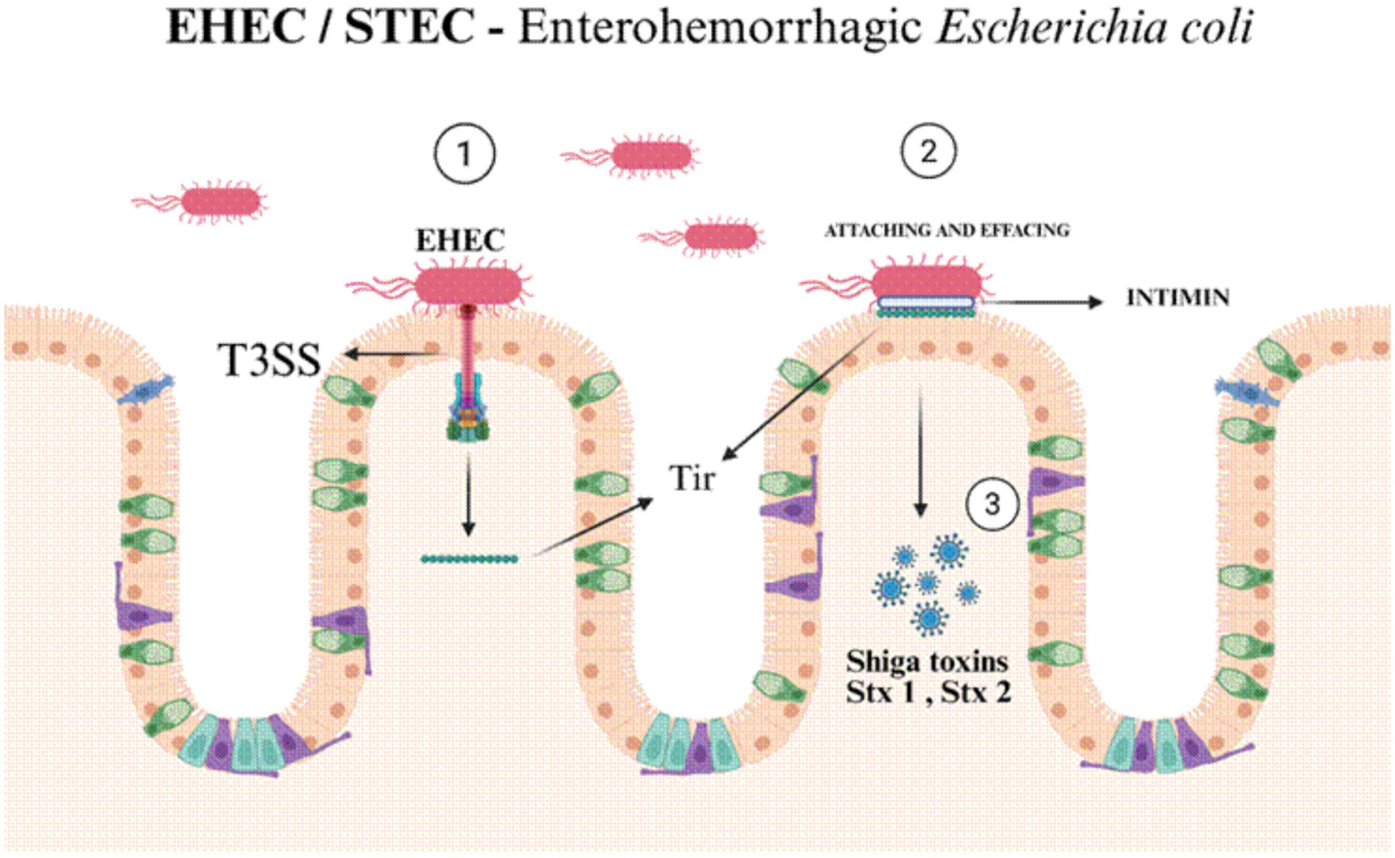
EHEC initially adheres to the intestinal surface using a type III secretion system (T3SS) (Step 1), which injects the translocated intimin receptor (Tir) into host enterocytes. The bacterium then binds to Tir via its outer membrane adhesin, intimin, resulting in intimate attachment and formation of attaching-and-effacing (A/E) lesions (Step 2), characterized by microvilli effacement and actin remodelling. Subsequently, the pathogen releases Shiga toxins (Stx1 and Stx2) (Step 3), which are internalised and inhibit host ribosomal function, leading to epithelial damage, hemorrhagic colitis, and in severe cases hemolytic uremic syndrome (HUS).

In Egypt and Iran, targeted testing of cattle and meat products has even identified multidrug-resistant STEC, raising concerns about how this pathogen may persist in the food supply over time (Heredia and García, 2018; Liu et al., 2022). Enterohemorrhagic *E. coli* (EHEC), another name for STEC, is considered a highly potent pathogen because of its ability to make Shiga toxins (Stx1 and/or Stx2) (Melton-Celsa, 2014). These toxins are encoded by prophages that become integrated into the bacterial genome. They belong to the AB5 toxin family and cause host cell death by halting protein synthesis (Melton-Celsa, 2014; Karmali et al., 2010). The toxins do this by cutting a specific site in ribosomal RNA, disrupting the ribosome’s role from being the protein-making machinery. Variants of Stx2 are especially linked to severe disease and are strongly associated with haemolytic uraemic syndrome (HUS), a life-threatening complication that results in haemolytic anaemia, low platelet counts, and acute kidney damage (Karmali et al., 2010; Tarr et al., 2005). Shiga toxins, many STEC strains including the O157: H7, as well as several non-O157 serogroups carry the LEE pathogenicity island. This genetic region enables them to create attaching and effacing lesions, that is similar to EPEC, which strengthens their ability to colonise and worsens mucosal injury (Croxen and Finlay, 2010) (Figure 5). Other virulence traits, such as enterohaemolysin which lyses red blood cells, further adds to the pathogenic potential.

### Clinical progression and severe complications

From a clinical perspective, STEC infections starts with watery diarrhoea which turns bloody (Table 1) within 3–4 days, reflecting colonic injury and bleeding (Croxen and Finlay, 2010; Karmali et al., 2010). Among infected children, about 5–15% progress to HUS, particularly if infected with Stx2-producing strains (Tarr et al., 2005). Contamination can occur during slaughter or through produce irrigated with contaminated water, making food chain a critical route of spread (Liu et al., 2022; Ren et al., 2024; Heredia and García, 2018).

### Diagnosis and surveillance challenges

Laboratory diagnosis requires specific detection of Shiga toxin genes (*stx1*, *stx2*) or their products cannot reliably separate STEC from harmless *E. coli* since it is ordinary culture method. PCR and enzyme immunoassays are now widely applied, and the advent of whole-genome sequencing has greatly improved outbreak investigations by identifying virulence gene profiles and phylogenetic links between isolates (Hussein, 2007). However, resource-limited regions often cannot apply these molecular tools, which contributes to the under-reporting of STEC in LMICs (Croxen and Finlay, 2010; Hussein, 2007).

### Treatment strategies and antimicrobial resistance concerns

Treatment is primarily supportive and focuses on hydration and complication management. Antibiotics are generally avoided as some can induce bacteriophage lysis and can cause increased toxin release, heightening the risk of HUS (Tarr et al., 2005; Croxen and Finlay, 2010). When HUS develops, dialysis and other supportive measures are required. Preventive strategies therefore focus on food safety, meat inspection, pasteurisation, and improved water hygiene remain the backbone of control (Brooks et al., 2005; Croxen and Finlay, 2010).

The rise of antimicrobial resistance (AMR) in STEC, that is particularly multidrug-resistant strains isolated from humans, cattle, and meat products in LMICs, adds another challenge (Heredia and García, 2018; Liu et al., 2022; Hussein, 2007). These isolates often carry plasmids combining virulence and resistance genes, which enables them to persist in the food chain and even transfer traits to other pathogens (Hussein, 2007). Despite progress in experimental vaccine development, no licensed human vaccine is yet available.

Promising efforts are underway in cattle, the main reservoir, where immunisation has been shown to reduce O157: H7 shedding and thus indirectly lower human risk (Brooks et al., 2005). In summary, STEC stands out as a zoonotic food-borne pathogen of major clinical and public health significance. Its ability to cause both acute bloody diarrhoea and severe sequelae such as HUS, together with its widespread presence in livestock, increasing recognition of non-O157 serogroups, diagnostic limitations in LMICs, and the mounting problem of AMR, highlights the pressing need for integrated food safety, One Health surveillance, and public health measures.

## EIEC

EIEC (Enteroinvasive *E. coli*) — EIEC has an invasive infection pattern similar to *Shigella*, relying on invasion plasmid genes such as *ipaH* to penetrate colonic epithelial cells. This results in a dysentery-like presentation, with symptoms including fever, abdominal cramping, and bloody stools. Although less frequently recovered in routine monitoring, EIEC occasionally appears in documented foodborne outbreaks (Heredia and García, 2018; Pasqua et al., 2017).

### Prevalence and outbreak occurrence

Across surveillance studies, Enteroinvasive *Escherichia coli* (EIEC) is usually detected only rarely with the prevalence rates in clinical panels often falling below 5%. Despite the low frequency, it still has the potential to spark localised outbreaks, and is often linked to contaminated vegetables or failures in sanitation systems (Acosta et al., 2016; Heredia and García, 2018). These sporadic events, although uncommon, serve as reminders that EIEC continues to play a role in dysentery-type illness.

### Invasive pathogenic mechanism

**Figure 6:**
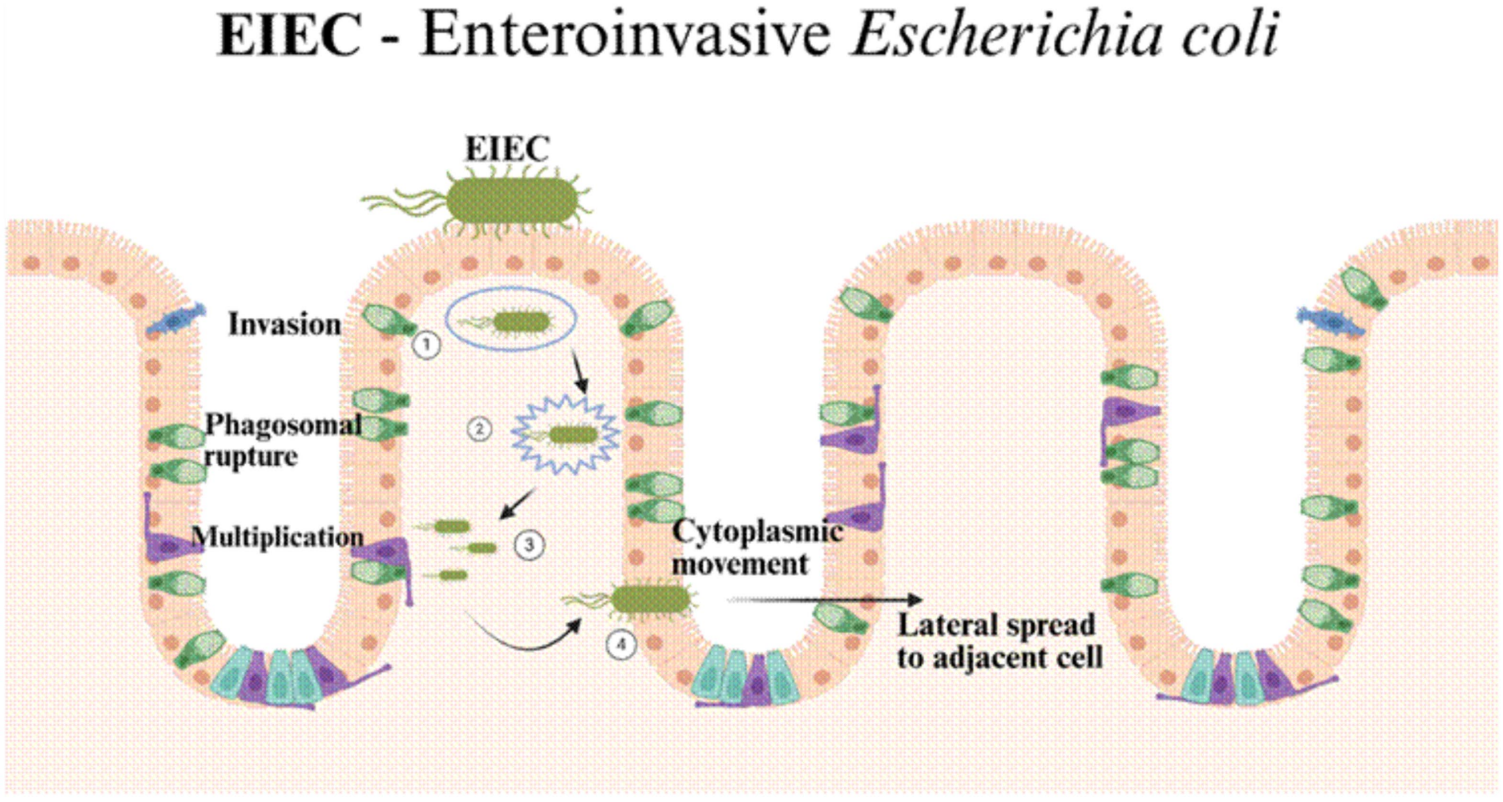
EIEC initiates infection by invading colonic epithelial cells through a type III secretion system-like mechanism, leading to uptake into a membrane-bound phagosome (Step 1). The bacterium subsequently escapes from the phagosome into the cytoplasm (Step 2), where it replicates intracellularly (Step 3). Actin-based motility enables cytoplasmic movement and lateral dissemination into adjacent epithelial cells (Step 4), resulting in epithelial destruction, mucosal ulceration, and dysentery-like inflammatory diarrhoea.

EIEC was first described in the mid-20th century, when strains resembling harmless *E. coli* biochemically were shown to cause illness almost identical to shigellosis. Genetic work has since confirmed that EIEC is very closely related to *Shigella*, with the two groups sharing much of their virulence machinery (Lan et al., 2004). Unlike other diarrhoeagenic *E. coli* that depend primarily on toxins or adherence factors, EIEC is notable for its invasive behaviour and ability to live and multiply inside host cells. The main determinant of this invasive capacity is the invasion plasmid (pINV), which carries genes such as *ipaH*, *ipaB*, *ipaC*, and *ipaD*, along with blueprint for a complete type III secretion system (T3SS) (Lan et al., 2004; Parsot, 2009). This secretion system functions as a molecular syringe, delivering bacterial effector proteins into host epithelial cells to disrupt the defenses and facilitate bacterial entry (Parsot, 2009) (Figure 6). Once inside, the bacteria break out of the vacuole, multiply in the cytoplasm, and then spread from one cell to another using actin-based motility a strategy nearly identical to that of *Shigella flexneri* (Sansonetti et al., 1982). The outcome is ulceration of the mucosa, infiltration of immune cells, and tissue damage.

### Clinical manifestations

The illness caused by EIEC mirrors this pathogenesis. Patients typically present with a dysentery-like syndrome characterized by fever, abdominal cramps, urgency, and diarrhoea that often contains mucus and blood (Lan et al., 2004). In contrast to secretory diarrhoeas caused by toxins, such as in ETEC, EIEC disease reflects genuine inflammation and destruction of colonic tissue (Nataro and Kaper, 1998). Although the disease is often self-limiting, vulnerable groups such as infants, older adults, and immunocompromised individuals can experience severe dehydration and complications.

### Transmission routes and food contamination

While less common than other DEC pathotypes, EIEC outbreaks continue to appear worldwide. Outbreaks are most frequently tied to contaminated water, inadequate hygiene or poor sanitation in LMICs (Acosta et al., 2016; Heredia and García, 2018). In higher-income settings, they have been associated with imported vegetables and ready-to-eat foods such as salads and carrots, showing how globalised supply chains can provide new routes for spread (Heredia and García, 2018; van den Beld and Reubsaet, 2012). The capacity of EIEC to persist in fresh produce is particularly worrying, as even small bacterial doses can lead to symptomatic infection in susceptible hosts.

### Diagnosis and antimicrobial resistance

From an epidemiological perspective, the importance of EIEC has probably been underestimated because of its striking similarity to both *Shigella* spp. and non-pathogenic *E. coli* (Lan et al., 2004; van den Beld and Reubsaet, 2012). Standard culture techniques are of little value, since EIEC lacks distinctive biochemical markers. As a result, molecular methods are very essential. PCR detection of *ipaH* is the most dependable approach, since this gene is present in multiple copies on both plasmid and chromosome. Other markers such as *virF* and *ial* may also be used to increase diagnostic specificity (Vantarakis et al., 2000). More recently, whole-genome sequencing has helped clarify the evolutionary relationship between *Shigella* and EIEC and has become a powerful tool in outbreak investigations to confirm strain identity and track spread (Lan et al., 2004; van den Beld and Reubsaet, 2012). Management of EIEC is mainly supportive with fluid and electrolyte replacement which is the cornerstone of therapy. Antibiotics can shorten the duration of illness but are generally reserved for severe cases which owes to the growing problem of resistance (Lan et al., 2004; Nataro and Kaper, 1998). Notably, the resistance has been reported to first-line drugs including ampicillin, trimethoprim-sulfamethoxazole, and fluoroquinolones. These trends closely parallel the situation in *Shigella* and are thought to arise from plasmid-mediated horizontal transfer of the resistance genes (Chellapandi et al., 2017). From a One Health perspective, the close genetic ties between EIEC and *Shigella*, which is combined with the ability to acquire resistance determinants from other enteric bacteria in humans, animals, and the environment, suggest that this pathotype may be an underestimated contributor to the wider AMR crisis.

### Public health implications

In conclusion, even though EIEC is relatively rare among the diarrhoeagenic *E. coli* group, its invasive lifestyle, shigellosis-like presentation, and capacity to cause foodborne outbreaks mean that it cannot be overlooked. The dual challenges of accurate diagnosis and rising drug resistance highlight the need for stronger molecular surveillance and outbreak monitoring. Alongside these, improvements in sanitation, safer food production practices, and prudent antibiotic use remain essential to minimise its impact on human health.

## AIEC

AIEC (Adherent-Invasive *E. coli*) — Distinguished by its ability to both attach to and invade intestinal epithelial cells, AIEC can also replicate within macrophages. Its clinical importance lies mainly in inflammatory bowel disease, especially Crohn’s disease. Consequently, prevalence studies for AIEC are typically concentrated in IBD (Inflammatory bowel disease) patient groups rather than broader enteric infection surveys (Martinez-Medina et al., 2009; Perna et al., 2020). Current Global and Regional Prevalence Data (2022–2025) — Synthesis from Primary Research.

### Association with inflammatory bowel disease

Since AIEC research has been concentrated mainly in inflammatory bowel disease (IBD) cohorts particularly in patients with Crohn’s disease it rarely appears in acute-diarrhoea prevalence surveys. Molecular and culture-based studies consistently demonstrate higher rates of AIEC isolation in paediatric Crohn’s patients compared with healthy controls, but beyond such populations, detection remains low and AIEC is largely absent from general DEC surveillance reports (Martinez-Medina et al., 2009; Perna et al., 2020).

### Cellular invasion and intracellular survival

Unlike other DEC pathotypes that are defined by toxin production or epithelial adherence patterns, AIEC is unique for its ability to adhere to and invade intestinal epithelial cells while also persisting within immune cells. A defining trait of AIEC is its ability to invade intestinal epithelial cells by binding to the surface receptor CEACAM6, which is highly expressed in the ileal mucosa of individuals with Crohn’s disease (Carvalho et al., 2009). Once AIEC has entered the host cells it replicates and breaches the epithelial layer then persists and multiplies inside macrophage cells without killing them. It does this in order to remain hidden from the immune system, continue to cause an inflammatory response and therefore create a continued cycle of infection and immune response (Glasser et al., 2001).

### Inflammatory response and virulence adaptations

The ability of AIEC to invade epithelial layers, along with its ability to survive inside host cells for extended periods of time, provides the basis for a continued cycle of infection and immune response. The presence of AIEC in the host results in the production of pro-inflammatory cytokines (TNF-*α*, IL-6 and IL-1β) due to the bacterial activity at the site of infection, leading to increased mucosal inflammation common in Crohn’s disease (Palmela et al., 2018). Additionally, the AIEC strains contain the genes required to produce long polar fimbriae (lpf), outer membrane proteins and other stress response mechanisms allowing them to survive in the inflamed intestinal environment in the presence of bile salts and oxidative stress (Martinez-Medina et al., 2009; Dogan et al., 2013).

### Clinical relevance in Crohn’s disease

Clinically, AIEC is most commonly associated with chronic intestinal inflammation and while it may contribute to acute watery diarrhea, it is most often associated with Crohn’s disease, supported by several studies that demonstrate higher colonization rates of AIEC in ileal lesions compared to normal tissue, association of AIEC with granulomatous inflammation and association of AIEC colonization with increased severity of the disease (Carvalho et al., 2009; Martinez-Medina et al., 2009; Palmela et al., 2018; Perna et al., 2020). The effects of AIEC on the host are further demonstrated through mouse models that show that colonization of mice with AIEC results in an increase in the level of intestinal inflammation, particularly in mice with genetic deficiencies in autophagy or innate immunity (Dogan et al., 2013). While the contribution of AIEC to ulcerative colitis is less clear, its strong connection with Crohn’s disease makes it an important pathotype in the study of chronic enteric disorders.

### Diagnostic challenges

Detection of AIEC is challenging, since there is no single genetic marker that defines this group. A diagnosis of AIEC typically requires multiple testing methods in combination. These include an adherence/invasion test using an epithelial cell line, gentamicin-protection assay for confirmation of intracellular replication by the bacteria, and macrophage infection assay demonstrating survival and non-apoptotic induction (Glasser et al., 2001; Martinez-Medina et al., 2009). Whole-genome sequencing has demonstrated that AIEC strains are not members of a unique lineage however they appear to be diverse populations of *E. coli* with independent acquisition of properties that allow them to invade and persist within the gut (Carvalho et al., 2009).

### Treatment difficulties and emerging strategies

Treatment of AIEC infections also appears to be difficult as standard antibiotics can sometimes provide temporary reduction in load of bacteria however recurrence is common and antibiotic-resistant strains have been identified in Crohn’s disease patients receiving chronic treatment (Carvalho et al., 2009; Palmela et al., 2018). Some studies suggest that anti-TNF therapy can indirectly reduce AIEC colonisation by dampening inflammation, though this effect is inconsistent (Glasser et al., 2001; Martinez-Medina et al., 2009). Experimental approaches include targeting CEACAM6-mediated adherence, restoring autophagy pathways, or using probiotics and phage therapy to selectively suppress AIEC populations (Carvalho et al., 2009; Palmela et al., 2018).

### Public health significance

From a public health standpoint, the importance of AIEC lies not in community diarrhoea burden but in its contribution to chronic intestinal disease. Its ability to exploit the inflamed gut as a reservoir, combined with its resilience inside immune cells, makes it a formidable player in Crohn’s pathogenesis. Although absent from most DEC prevalence studies, AIEC continues to be a focus of IBD research, and controlling it may 1 day form part of targeted therapies for chronic inflammatory disorders.

### Epidemiological shifts in dominant pathotypes—key patterns and their significance

#### Increase in EAEC prevalence

The prevalence of EAEC has increased, which is a significant recent development. Hospital-based and community investigations have revealed that EAEC is increasingly challenging and sometimes surpassing ETEC and EPEC, which have historically dominated pediatric diarrheal illnesses. An assessment of primary research conducted between 2015 and 2025 makes it clear that the distribution of DEC pathotypes is shifting dramatically across a number of geographical areas (Singh et al., 2019).

#### Variation by surveillance setting and age group

Prevalence trends vary by age group and survey period, with hospital-based studies typically reporting higher proportions of severe pathotypes such as EHEC due to inclusion of more clinically serious cases, whereas birth-cohort and community surveillance studies more frequently identify high burdens of secretory pathotypes such as ETEC that cause milder but more frequent infections (Platts-Mills et al., 2015).

#### Emergence of hybrid and resistant strains

Hybrid strains and the growing number of antibiotic resistant strains make these issues worse. Recent isolates possess virulence factors from multiple DEC pathotypes, creating hybrid strains with complexity that blend traditional classification categories. These hybrid strains combined with increasing antibiotic resistance rates create additional challenges to control and monitor diseases (Singh et al., 2019; Liu et al., 2022).

#### Factors driving these changes-biological characteristics of EAEC

The organism’s function in chronic illness and its long-term nutritional influence on children is explained by its capacity to create strong intestinal biofilms and stay in the gut for longer periods of time. Because of these characteristics, it is an especially difficult target for prevention and treatment (Acosta et al., 2016; Singh et al., 2019).

#### Improvements in diagnostic capacity

Changes in laboratory capacity, particularly the introduction of multiplex PCR and whole-genome sequencing in routine diagnostics, are linked to this trend (Liu et al., 2022; Heredia and García, 2018; Ren et al., 2024).

#### Environmental and food-chain exposure

Environmental routes and the food chain are also becoming more thoroughly documented. STEC, ETEC, and EPEC contamination of meat and fresh vegetables has been proven by investigations, and these isolates frequently carry antimicrobial-resistance genes. These results are consistent with the One Health concept, which holds that environmental cleanliness, food processing, and cattle husbandry all work together to determine the risk of human exposure (Heredia and García, 2018; Ren et al., 2024; Liu et al., 2022). These differences reflect variations in disease severity, healthcare-seeking behaviour, and study design, which together influence which DEC strains appear dominant across different surveillance settings (Breurec et al., 2016).

Practical implications of these shifts include the urgent need for:

- Standardized molecular diagnostic techniques that enable direct comparisons between results from various places.
- To follow contamination routes more effectively, surveillance must unify data from human, animal, and environmental health sectors.
- Ongoing efforts to produce vaccines, starting with ETEC and moving on to other, promising candidates.
- When supported by reinforced antibiotic stewardship, they play a crucial role in curbing the spread of resistant DEC strains.

### Current drug resistance trends across pathotypes

#### Historical and evolving therapy context

Traditionally, ampicillin and trimethoprim-sulfamethoxazole (TMP-SMX) served as the leading oral treatments for many community-acquired diarrheal infections. For more severe cases or when first-line therapies failed, fluoroquinolones and third-generation cephalosporins became the second line of defense. However, over the past decade these well-established treatment paradigms have been disrupted by the rapid rise of resistance across various DEC pathotypes. This trend has compelled clinicians to rely increasingly on a narrower-spectrum or parenteral agents for severe infections which amplifies the need for antibiotic stewardship and tailored therapy guided by local susceptibility profiles (Baquero et al., 2014; Prestinaci et al., 2015; Liu et al., 2022; Qadri et al., 2005).

### Resistance patterns by drug class

#### Ampicillin

Ampicillin resistance remains broadly high among DEC and *Shigella* isolates. Multiple surveillance efforts at regional and multicenter levels report phenotypic resistance rates frequently exceeding 30–60% in both community and pediatric populations. This persistence stems mainly from long-standing selective pressures and the stable presence of beta-lactamase genes carried within enteric bacterial populations. Plasmid-mediated production of narrow-spectrum beta-lactamases (non-ESBL) and the co-selection of resistance genes on mobile genetic elements contribute heavily to ampicillin resistance being maintained among successful clones circulating in these infections (Prestinaci et al., 2015; Taha and Yassin, 2019; Cantón and Coque, 2006; Heredia and García, 2018; Carattoli, 2013).

### Trimethoprim–Sulfamethoxazole (TMP-SMX)

Resistance to TMP-SMX is similarly widespread and often co-occurs with ampicillin resistance which diminishes the reliability of this agent for empirical therapy in many settings. Surveillance data from community and pediatric cohorts underscore this trend with pooled analyses linking TMP-SMX resistance to integrons and class 1 mobile genetic elements that also carry the resistance genes for aminoglycosides and sulfonamides. Such genetic platforms reinforce the multidrug resistance (MDR) phenotypes seen in many clinical DEC isolates (Prestinaci et al., 2015; Singh et al., 2019; Qadri et al., 2005; Cantón and Coque, 2006; Heredia and García, 2018).

### Fluoroquinolones

Fluoroquinolone resistance has grown markedly in recent years, becoming commonplace within certain DEC lineages, extraintestinal *E. coli*, and *Shigella* strains. Resistance mechanisms include chromosomal mutations in quinolone-resistance determining regions (QRDR) and plasmid-borne qnr genes, with plasmid carriage accelerating resistance dissemination across diverse strains and pathotypes. Genomic surveillance highlights that fluoroquinolone resistance frequently clusters within MDR pandemic clones, such as the H30/H30-Rx lineages, with geographic hotspots shaped by regional antibiotic use practices (Baker et al., 2018; Liu et al., 2022; Qadri et al., 2005; Singh et al., 2019; Johnson et al., 2010).

### Azithromycin (Macrolides)

Azithromycin has gained favor as an oral agent for treating *Shigella* and some *E. coli* infections in community settings. Yet, over the past 5 to 7 years, surveillance and molecular studies have documented sharp increases in azithromycin reduced susceptibility and clinical resistance. This resistance is often linked to genes such as mph(A), erm, and efflux pump systems. High-resolution genomic analyses reveal global dissemination of azithromycin-resistant *Shigella* clones with several regions reporting abrupt post-pandemic surges in macrolide resistance. The mobile genetic nature of the macrolide resistance determinants raises concerns about rapid horizontal transfer to other species within enteric microbiota (Liu et al., 2022; Baker et al., 2018; Ingle et al., 2019; DuPont, 2015; Qadri et al., 2005).

### Third-generation cephalosporins/ESBLs

Resistance to third-generation cephalosporins has escalated,which is largely driven by the spread of extended-spectrum *β*-lactamases (ESBL) especially CTX-M enzymes among DEC pathotypes including ETEC, EPEC, and EAEC. Growing regional evidence from genomic and phenotypic surveys underscores the high prevalence of ESBL producers, diminishing the effectiveness of oral cephalosporins and prompts frequent escalation to carbapenem therapy in severe or invasive infections. The epidemic spread of *CTX-M*-carrying plasmids contributes to the rapid geographic dissemination and co-resistance to other antibiotic classes when these plasmids harbor multiple resistance genes (Qadri et al., 2005; Taha and Yassin, 2019; Cantón and Coque, 2006; Heredia and García, 2018; Carattoli, 2013).

### Rifaximin

Owing to its minimal systemic absorption, rifaximin has been a favored option for traveler’s diarrhea and localized gut infections,on the assumption that it induces limited systemic resistance. However, newer experimental and genomic data challenge this view, showing that rifaximin use can select for resistance elements within the gut microbiome that also promote cross-resistance or co-selection of resistance to critical systemic antibiotics among commensal and opportunistic bacteria. However, rifaximin resistance among typical enteric pathogens remains less studied when compared to macrolides or cephalosporins, caution while using is warranted given its potential to drive resistance selection pressures (Baquero et al., 2014; DuPont, 2015).

### Pathotype-specific resistance and MDR trends

- ETEC: Once largely susceptible to older antimicrobials, ETEC is exhibiting rising multidrug resistance and ESBL production in certain regions, which severely limits the available oral treatment options. ESBL-positive ETEC strain reports have increased from pediatric cohorts which signals heightened concerns (Taha and Yassin, 2019; Singh et al., 2019; Qadri et al., 2005).
- EPEC/EAEC: High rates of ampicillin and TMP-SMX resistance are common in these pathotypes (Table 3) where EAEC is often linked to particularly high MDR prevalence in some regional studies (Prestinaci et al., 2015; Singh et al., 2019).
- EHEC/STEC: Antibiotic use is complicated when given the risk of hemolytic-uremic syndrome (HUS) in STEC infections is present. Resistance surveillance focuses on fluoroquinolones and cephalosporins where antibiotics are indicated for systemic issues. Genomic analyses reveal the emerging resistant subclones based on plasmid and lineage data (Liu et al., 2022; Qadri et al., 2005).
- EIEC: EIEC shows some of the most alarming resistance trends, particularly to macrolides and fluoroquinolones. Epidemic clones carrying mph (A), qnr, and ESBL genes are spreading internationally which contributes to extensively drug-resistant (XDR) clusters that are identified in multiple studies (Baker et al., 2018; Ingle et al., 2019; DuPont, 2015).
- AIEC: Pandemic extraintestinal *E. coli* clones such as ST131 and emerging ST410 display high fluoroquinolone and cephalosporin resistance rates with recent acquisition of carbapenemase genes. This shift represents an evolution towards severe MDR phenotypes which blends enteric and extraintestinal disease risks (Singh et al., 2019; Johnson et al., 2010; Liu et al., 2022). Genomic evidence highlights that the epidemic plasmids and successful clonal lineages including H30/H30-Rx, ST131, ST410 and resistant *Shigella* clusters are central drivers sustaining a contemporary MDR profiles by facilitating rapid acquisition and global spread of multiple resistance determinants simultaneously (Singh et al., 2019; Liu et al., 2022; Qadri et al., 2005; Carattoli, 2013).

**Table 3:** Antibiotic classes, mechanisms of antimicrobial resistance, and *e. coli* pathotypes commonly exhibiting resistance.

Antibiotics class	Antimicrobial resistance mechanism	Common drugs used	Pathotypes commonly showing resistance
β-lactams (Penicillins, Cephalosporins, Carbapenems)	Production of β-lactamases (e.g., *blaTEM, blaSHV, blaCTX-M*); Altered PBPs; Porin loss	Amoxicillin-clavulanate, Ceftriaxone, Cefotaxime, Meropenem	ETEC, EPEC, EAEC, STEC, EIEC, AIEC
Tetracyclines	Efflux pumps (*tetA, tetB*) and ribosomal protection proteins	Doxycycline, Minocycline	ETEC, EPEC, EAEC, EIEC
Sulfonamides & Trimethoprim	Target enzyme modification (*sul1, sul2, dfrA*) reduces binding to folate pathway enzymes	Cotrimoxazole (Trimethoprim-sulfamethoxazole)	ETEC, EPEC, EAEC, EIEC, AIEC
Aminoglycosides	Aminoglycoside-modifying enzymes (*aadA, aac(3)-IIa*) and efflux	Gentamicin, Amikacin	EAEC, STEC, AIEC
Fluoroquinolones (Quinolones)	Mutations in *gyrA/parC* (DNA gyrase/topoisomerase IV) and *qnr* plasmid genes	Ciprofloxacin, Levofloxacin, Norfloxacin	EPEC, EAEC, STEC, AIEC
Macrolides	Methylation of 23S rRNA (*ermB, mphA*) prevents drug binding	Azithromycin	EAEC, ETEC
Chloramphenicol	Enzymatic inactivation by catA (chloramphenicol acetyltransferase)	Rarely used; alternative agents preferred	EPEC, EIEC
Carbapenems (Reserve drugs)	Carbapenemase production (*blaNDM*, *blaOXA-48*)	Meropenem, Imipenem	AIEC (especially clinical isolates)

Source: Morita and Kuroda (2025), Nasrollahian et al. (2024).

### Clinical and public health implications

Empirical treatment of diarrhea in the community should always consider up-to-date local antimicrobial susceptibility data, as historical first-line agents such as ampicillin and TMP-SMX have become unreliable alternatives in many areas (Prestinaci et al., 2015; Heredia and García, 2018; Singh et al., 2019). The increasing resistance to azithromycin and fluoroquinolones, coupled with expanding ESBL presence, limits oral treatment options for outpatient care and shifts the therapeutic demand towards parenteral agents in severe infections (Ingle et al., 2019; Qadri et al., 2005). Vigorous antibiotic management is crucial. This includes judicious use of rifaximin which restricts the unnecessary macrolide exposure, and limits fluoroquinolone usage to prevent further selection of resistant strains. Complementary to the management, routine genomic surveillance to track plasmid-mediated resistance and epidemic clone emergence is essential for effective containment of these strategies (Baquero et al., 2014; DuPont, 2015; Cantón and Coque, 2006; Carattoli, 2013).

### Mechanisms of antimicrobial resistance in diarrheagenic *Escherichia coli*

**Figure 7:**
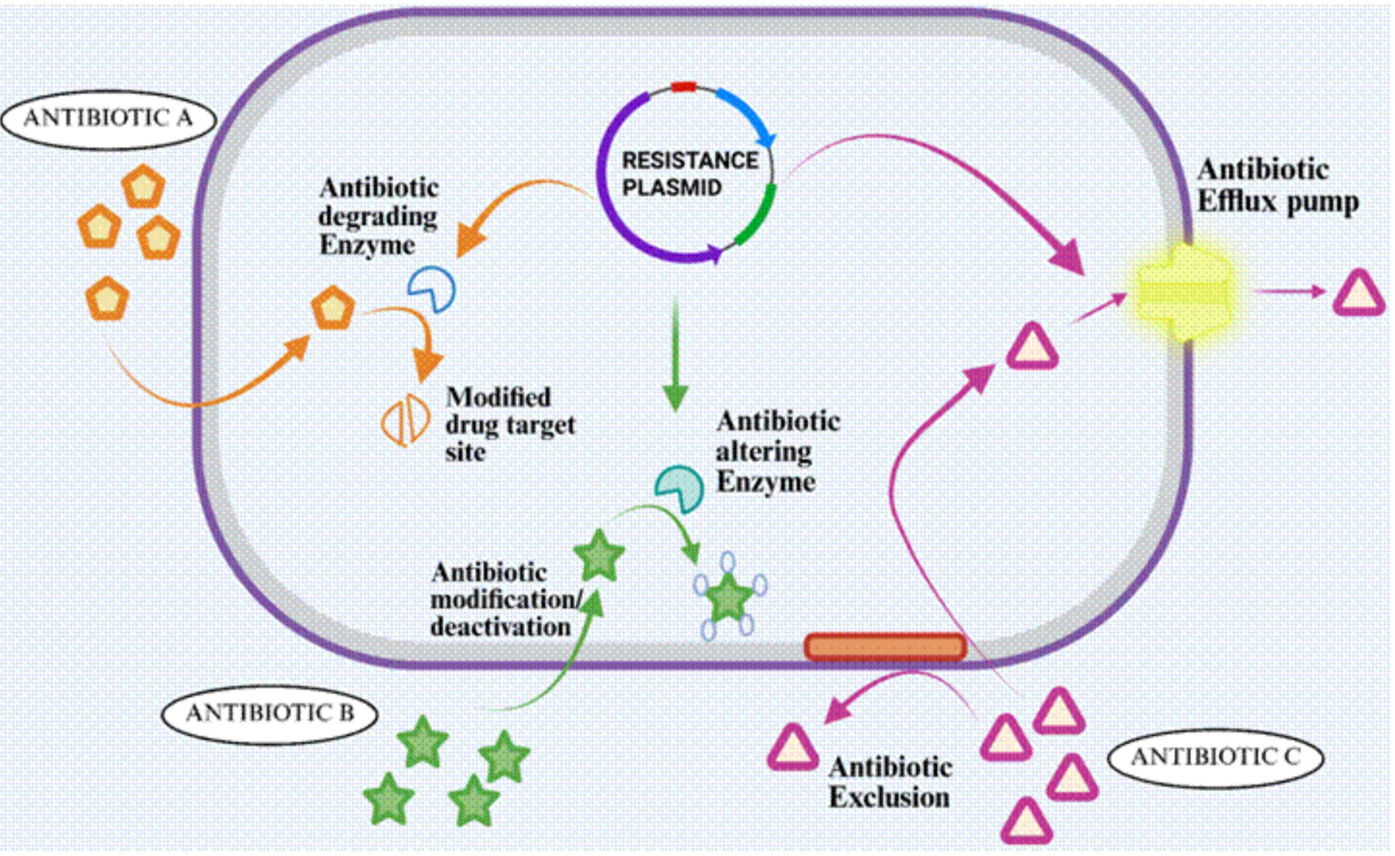
Mechanisms of antimicrobial resistance in diarrheagenic *Escherichia coli.*

DEC is equipped with a wide collection of molecular defenses to withstand antibiotic treatment. Resistance across major pathotypes including EPEC, ETEC, EAEC, EIEC, and EHEC arises through several complementary strategies. These include the production of inactivating enzymes, use of efflux pumps and reduced membrane permeability, biofilm–associated protection and the acquisition of mobile genetic elements (Figure 7). These mechanisms drive the high resistance rates observed in clinical and environmental DEC isolates together.

#### Enzymatic inactivation

A typical pattern among the resistant DEC strains is their ability to directly neutralize antibiotics. Many *E. coli* strains produce enzymes that neutralize antibiotics through hydrolysis or other chemical modifications. Among the most significant are the extended-spectrum *β*-lactamases (ESBLs), including the CTX-M, TEM and SHV families, which can inactivate third-generation cephalosporins. These resistance genes are frequently located on transferable plasmids, allowing them to move easily between bacteria and rapidly amplify their spread in microbial communities (Cantón and Coque, 2006). Aminoglycoside resistance frequently stems from modifying enzymes like acetyltransferases and phosphotransferases; these have been documented in both animal and human sources, where they alter the antibiotic’s structure and protect the ribosome from inhibition (Liu et al., 2022; Singh et al., 2019). On a different front, disruptions to central metabolic enzymes such as those involved in glycolysis or the TCA cycle have also emerged as a surprising route to multi-drug tolerance, with targeted inactivation leading to increased antibiotic survival in experimental models (Baquero et al., 2014).

#### Efflux pumps and decreased permeability

Limiting the antibiotic entry and actively pumping drugs out of the cell is another critical resistance mechanism. DEC isolates commonly utilize energy-dependent efflux pumps such as the AcrAB–TolC system (part of the RND family), which has been tied to a decreased susceptibility to agents like fluoroquinolones and tetracyclines even when classical resistance genes are not detected (Baquero et al., 2014; Singh et al., 2019; Cantón and Coque, 2006). This efflux is often paired with lower permeability due to changes in major porins, including OmpF and OmpC. Clinical isolates harboring resistance genes or ESBLs frequently display downregulation or structural alterations of these porins,which restrict drug uptake and boost resistance beyond that conferred by efflux alone (Baquero et al., 2014; Singh et al., 2019; Tadesse et al., 2012). While detailed studies of mutations in global regulators like *marA* or acrR in DEC remain scarce, current evidence suggests that such regulatory changes further potentiate these combined efflux–permeability defenses (Liu et al., 2022).

#### Role of biofilms

Biofilm formation renders DEC even more resilient in the face of antibiotic stress. When strains like EAEC and EPEC establish strong biofilms, antibiotic penetration is hampered by the extracellular polymeric matrix which also helps shield embedded bacteria from host immune responses (Liu et al., 2022; Saldaña et al., 2009; Cantón and Coque, 2006). Animal studies confirm that EPEC can produce biofilm-like communities within the intestine which lead not only to tissue damage but also persistent infection and altered host metabolism (Liu et al., 2022; Saldaña et al., 2009). These microenvironments also allow some cells, so called persisters, to survive otherwise lethal drug concentrations. The dense biofilm structure provides an ideal setting for horizontal gene transfer to flourish among co-inhabiting DEC cells additionally (Liu et al., 2022; Saldaña et al., 2009; Baquero et al., 2014).

#### Horizontal gene transfer (HGT) and mobile elements

The spread of resistance in DEC is driven mainly by horizontal gene transfer. Field studies from Nigeria and Delhi have shown a high frequency of class 1 integrons that carry resistance cassettes such as *dfrA*, which provides resistance to trimethoprim, and *aadA*, which confers resistance to aminoglycosides (Baquero et al., 2014; Odumosu et al., 2013; Rawat and Nair, 2010). These integrons, often located on conjugative plasmids, promote the transfer of resistance genes not only among DEC strains but also across other enteric bacteria, greatly increasing their adaptive potential (Baquero et al., 2014; Odumosu et al., 2013). Alongside this, mobile genetic elements such as transposons and insertion sequences particularly IS26 mobilize *β*-lactamase genes and other resistance traits, enabling the formation of complex multidrug resistance islands (He et al., 2015). Studies of both animal and human sources including calves and international travelers support that resistance plasmids frequently move between animal reservoirs and human DEC strains, highlighting the global and zoonotic dimensions of HGT-driven resistance (He et al., 2015; Singh et al., 2019; Tadesse et al., 2012; Treacy et al., 2019).

### Integrative perspective

Rarely do resistance mechanisms in DEC operate independently. Extensive multidrug resistance may arise from a single strain that concurrently produces ESBLs, upregulates efflux pumps, and downregulates porins. By promoting intimate connections and enabling horizontal gene transfer, biofilms exacerbate this issue by often connecting virulence and resistance determinants on shared mobile elements. Improving stewardship procedures and directing the creation of new treatments require an understanding of how these systems interact.

### Emerging technologies which can combat AMR

Therapeutic development approaches have changed as a result of the ongoing global burden of diarrheagenic *E. coli* (DEC) and other intestinal bacteria that are resistant to drugs. In order to supplement or eventually replace traditional antibiotics, emerging approaches currently prioritize microbiome-centered, technology-integrated, and nature-inspired therapies. Currently, a broad spectrum of innovative approaches is being explored to counter antimicrobial resistance (AMR). These include phage therapy, vaccine development, antimicrobial peptides, nanotechnology applications, CRISPR-based gene editing, disruption of bacterial communication pathways, and monoclonal antibody strategies (Jacobowski et al., 2025; Carnero Canales et al., 2025; de Oliveira et al., 2024; Huang et al., 2024). These approaches, especially when combined with restoring the gut microbiome, provide a complete strategy to reduce global risks from drug-resistant infections.

#### Phage-based therapies

Phage therapy is back as a precise way to target MDR infections. Thanks to modern gene sequencing, computer screening, and lab-made phage design, these viruses are now much more specific to their hosts and their treatment is more predictable (Jacobowski et al., 2025; Carnero Canales et al., 2025). In studies of diarrheagenic *E. coli* (DEC), phage mixtures targeting EAEC, ETEC, and EPEC have been able to destroy sticky bacterial layers (biofilms) and lower harmful bacteria without affecting beneficial gut microbes (Karami et al., 2024; Xiong et al., 2024). Using them alongside antibiotics makes them even stronger: pre-exposing the bacteria to antibiotics weakens the biofilm, letting the phages sneak deeper inside (Karami et al., 2024). Recent breakthroughs in delivery, like wrapping phages in tiny carriers made of chitosan or liposomal nanoparticles, have made phages more stable, better able to survive stomach acid, and allowed for controlled release in the intestines (Jacobowski et al., 2025; Huang et al., 2024). “Smart phages” have even been engineered to carry CRISPR payloads that can selectively cut out resistance genes like *bla*(CTX-M) or *mecA*, combining germ-killing with gene-editing power (Jacobowski et al., 2025). These hybrid phage systems are a game-changer in treatments we can biologically program.

#### Vaccine development

Of all the DEC types, ETEC is the main focus for vaccines because its targets are somewhat predictable and it causes so much illness. Subunit vaccines that use colonization factors (CFs), multi-part structures and detoxified toxin antigens have reached late-stage clinical trials (Phase 2–3), showing over 50% protection against moderate-to-severe diarrhoea (Khalil et al., 2021; Svennerholm and Lundgren, 2012). For EPEC and EAEC vaccines, scientists are focusing on common sticky proteins like intimin (Oswald et al., 2000; Svennerholm and Lundgren, 2012; McKee et al., 1995). New work in immuno-nanotechnology uses tiny protein or nanoparticle frames to improve how the vaccine is delivered to the gut lining and boost immune memory, which helps solve the problem of oral vaccines breaking down quickly in hot climates (Jacobowski et al., 2025; Huang et al., 2024). For STEC, detoxified Shiga-toxin antigens are still being tested, requiring very careful safety checks. All these steps show how tweaking antigens and using tiny delivery systems (nanoplatforms) can significantly strengthen the pipeline for gut vaccines.

#### Antimicrobial peptides (AMPs)

AMPs (Antimicrobial Peptides) are gaining recognition as promising biological drugs because they kill many types of germs, have a low chance of creating cross-resistance and can also help modulate the immune system in various ways (Jacobowski et al., 2025; de Oliveira et al., 2024). Lab-modified versions using D-amino acids, circular structures, or combined sequences are now better at resisting breakdown by enzymes and at targeting specific bacteria. When the improved versions of LL-37 and piscidin have been studied they have shown that they are effective against *P. aeruginosa* biofilms and *E. coli* and causing minimal harm to human cells (Jacobowski et al., 2025; Carnero Canales et al., 2025). Attaching antimicrobial peptides (AMPs) to nanocarriers has greatly improved their delivery, helping them reach the target site more efficiently and release their effects directly in the gut (Carnero Canales et al., 2025; Huang et al., 2024). Gold and polymer-based nanoparticles loaded with AMPs work together to destroy harmful bacteria and prevent biofilm formation (Carnero Canales et al., 2025). This shows strong potential for use along with phages or antibiotics. AMP-based products are now becoming leading options to fight drug-resistant bacteria (de Oliveira et al., 2024).

#### Anti-virulence strategies

These treatments aim to weaken harmful microbes instead of killing them directly, which helps prevent the development of antibiotic resistance (de Oliveira et al., 2024; Fleitas Martínez et al., 2019; Laxminarayan et al., 2016). Molecules that stop the bacterial communication known as quorum-sensing, block the type III secretion system, or inhibit adhesins are showing good results in early-stage models (Jacobowski et al., 2025). New approaches combine AMPs or small molecules that quench quorum-sensing to stop the production of virulence factors, block biofilm formation, and prevent the spread of resistance genes (de Oliveira et al., 2024; Jacobowski et al., 2025). By making the bacteria less harmful without affecting their survival, these treatments act as a partner to existing antibiotics and help maintain the natural balance of the gut microbiome.

#### Microbiota modulation

The gut microbiome is key to stopping DEC from taking hold and plays a vital role in immune responses. Probiotics like *Lactobacillus rhamnosus* GG and *Saccharomyces boulardii* compete with pathogens, strengthen the gut barrier, and fire up the mucosal immune system (Hill et al., 2014; Guarino et al., 2015). Recent integrated strategies combine probiotics with AMP- or phage-based therapies, using competition and immune boosting for long-term control of colonization (Jacobowski et al., 2025). Faecal Microbiota Transplantation (FMT) continues to show promise in fixing the gut imbalance (dysbiosis) that follows infection or antibiotic use, though we still need to standardize donor screening and delivery methods (Carnero Canales et al., 2025; Jacobowski et al., 2025).

#### Monoclonal antibodies (mAbs)

Monoclonal antibodies (mAbs) offer a targeted way to neutralize DEC toxins and adhesion molecules. Covering antibodies with protective coatings or placing them inside capsules allows them to be taken by mouth, as this shields them from being destroyed by stomach acid (Luiz et al., 2015; Sheoran et al., 2003). Recent progress in developing specialized antibodies, such as bispecific and recombinant secretory IgA, allows them to target multiple harmful factors at once and stay active longer in the gut. When used alongside vaccines or other gut-focused treatments, these antibodies can help both prevent and treat drug-resistant intestinal infections.

#### Nanotechnology-enabled therapeutics

Nanotechnology links accurate diagnosis with flexible treatment options, combining materials science with microbiology. Metallic (Ag, Au, ZnO) and polymeric (PLGA, chitosan) nanoparticles show inherent germ-killing effects by disrupting cell membranes, causing oxidative stress, and blocking efflux pumps (Jacobowski et al., 2025; Huang et al., 2024). Adding peptides or antibodies makes them more specific while reducing overall toxicity. Hybrid nanocomposites like phage-chlorin e6-MnO₂ systems combine light-activated (photodynamic) and enzyme-based germ-killing actions to wipe out biofilms (Huang et al., 2024). These tiny platforms demonstrate how controlled-release and ‘smart’ nanomaterials that respond to stimuli can get around classic resistance mechanisms.

#### CRISPR-Cas systems

CRISPR-Cas tools bring a genetic-level approach to tackling AMR. Programmable tools like Cas9 and Cas13 can be used to cut or switch off resistance genes, helping old antibiotics work again (Jacobowski et al., 2025). Using phages to deliver the CRISPR system makes sure it attacks only the resistant bacteria, leaving the helpful gut bacteria unharmed. Scientists are also testing ways to deliver CRISPR into gut bacteria using tiny fat-based particles (lipid nanoparticles) or beneficial bacteria such as Lactobacillus that can carry the CRISPR material (Huang et al., 2024). In addition, CRISPR interference (CRISPRi) can block the genes that bacteria use to communicate with each other, which helps stop the formation of biofilms in bacteria like *Pseudomonas aeruginosa*. Antimicrobials based on CRISPR provide us with a groundbreaking approach to treatments that could potentially be immune to resistance.

#### Quorum-sensing inhibition

Quorum-sensing (QS) interference damages the bacterial communication systems which are responsible for management of virulence and resistance. Small molecules, such as halogenated furanones, chalcone derivatives, and flavonoids, competitively block the signals (autoinducers) from being made or from binding to their receptors (Jacobowski et al., 2025; Carnero Canales et al., 2025). Enzymatic QS disruption using enzymes like lactonases or acylases breaks down the integrity of biofilms and restores sensitivity to antibiotics (Jacobowski et al., 2025). These QS-focused methods fit the anti-virulence philosophy: they reduce a pathogen’s power without increasing the pressure for it to evolve stronger resistance.

#### Integrated and translational outlook

While no single new technology can take the place of antibiotics on its own, their combined use provides a potent and flexible approach to combating antimicrobial resistance. For the treatment of complicated infections, the combined use of bacteriophages, antimicrobial peptides, nanotechnology-based delivery methods, CRISPR-mediated tools, and immunotherapies shows significant potential. Strong interdisciplinary cooperation will be necessary to make these breakthroughs scalable, affordable, and accessible in order to sustain progress, especially in low- and middle-income nations where AMR is the biggest problem.

### Emerging omics insights in antimicrobial resistance in diarrheagenic *Escherichia coli* (DEC)

#### Genomic epidemiology and evolution of AMR determinants in diarrheagenic *E. coli* (DEC)

Antimicrobial resistance (AMR) in diarrheagenic *E. coli* (DEC) is now understood from a complex One-Health genomic ecology where resistance traits are constantly exchanged between human, animal, food and environmental reservoirs. Instead of relying solely on conventional clonal propagation, high-throughput surveillance shows that conjugative plasmids, integrons, insertion sequences and phage-associated cargo play a major role in the spread of resistance in DEC (Hoshiko et al., 2025; Sung et al., 2024). Extended-spectrum *β*-lactamases (ESBLs), plasmid-mediated quinolone resistance determinants, and multi-drug efflux systems are carried by these plasmids, especially IncF, IncI, and IncX, creating a highly mobile resistome that crosses ecological barriers (Kheiri and Akhtari, 2016; Santos et al., 2020). Genome-resolved metagenomics from wastewater, livestock operations, fresh produce, and surface water sources show shared AMR signatures between human isolates and environmental microbiomes which supports the concept of ecological recycling and environmental amplification of clinical resistance determinants (Akinlabi et al., 2025; Abdalla et al., 2025). Environmental stressors which include disinfectant residues, heavy metals, and agricultural antibiotics, act as co-selection pressures, enriching plasmid-encoded resistance clusters and driving persistence in non-clinical niches (Muloi et al., 2022; Narwal et al., 2025). An important upcoming trend is the merging of virulence and resistance, where hybrid DEC strains carry overlapping EAEC, EPEC, and ETEC virulence loci alongside strong AMR determinants, producing high-risk genotypes with dual pathogenic and resistant phenotypes (Bielaszewska et al., 2007; Finton et al., 2025). Long-read sequencing has been involved in mapping these mosaic plasmid-virulence architectures, revealing the circulation of nearly identical virulence-resistance plasmids between pediatric diarrheal cases and poultry/wastewater sources (Skarlatoudi et al., 2025; Srinivasan et al., 2025). The evolutionary success of these hybrid DEC lines explains that AMR is now coiled with virulence evolution and ecological fitness, reshaping our view of enteric pathogen emergence (Santos et al., 2020; Srinivasan et al., 2025; Nataro and Kaper, 1998).

#### Pan-genomics and comparative genomics of DEC pathotypes

Pan-genomic analysis shows that *E. coli* has a vast, open and expandable pan-genome, the accessory gene pool exceeds core genomic elements and acquires new functions through horizontal gene transfer (Rasko et al., 2008; Wang et al., 2023). DEC pathotypes hold on to diverse accessory islands encoding adhesins, biofilm regulators, iron acquisition systems, secretion systems, metabolic adaptation genes and antibiotic tolerance modules, enabling flexible pathogenic and survival strategies across hosts and environments (Du et al., 2024; Kumar et al., 2025). Comparative genomics shows that traditional pathotype classifications deteriorate as hybrid strains overlap virulence systems, unfocusing boundaries between EAEC, EPEC, and ETEC groups (Ortega-Balleza et al., 2023; Finton et al., 2025). Such genomic plasticity allows DEC to change between virulence lifestyles and environmental survival states, increasing their persistence across agricultural settings, sewage infrastructure, and clinical reservoirs (Swift et al., 2019; Hotez and Kamath, 2009). Pangenome-wide association studies (Pan-GWAS) combined with machine learning are identifying conserved antigenic and metabolic nodes across diverse DEC genomes, supporting precision vaccine development and next-generation drug target discovery. These analyses reveal plasmid-encoded metabolic fitness traits like iron uptake and biofilm enhancers co-selected along with AMR, fortifying the concept that antibiotic resistance evolution is coupled with ecological adaptation (Saini et al., 2024; Xu et al., 2025).

#### Artificial intelligence and machine learning in AMR prediction

Machine learning (ML) and artificial intelligence (AI) offer extremely potent methods for tracking epidemics, warning systems, and genotype-to-phenotype AMR prediction. Beyond rule-based ARG detection alone, models trained on k-mer signatures, pangenome presence/absence profiles, plasmid replicons, integron cassettes, and regulatory mutations accurately predict AMR symptoms and multidrug resistance risk in *E. coli* (Mediouni et al., 2025). In order to ensure interpretability and clinical trust, AI techniques like SHAP interpretation and feature-ranking identify the causative genetic determinants driving resistance results, such as *blaCTX-M* context, *qnr* variations, porin mutations, and efflux regulatory circuits (Aytan-Aktug et al., 2021; Kim et al., 2022; Mediouni et al., 2025). The multi-label machine learning approach makes it possible to predict resistance to several antimicrobials and to adapt to different host species, geographical locations, and environmental conditions. AI systems are also advancing in antimicrobial discovery by mining microbiome and metagenomic databases for novel antimicrobial peptides and anti-virulence compounds, identifying structures beyond human intuition and accelerating drug-candidate generation pipelines (Santos-Júnior et al., 2023). Clinically, AI-assisted workflows integrated with diagnostic laboratories give rapid risk alerts for resistant DEC strains before routine susceptibility testing is completed, strengthening infection control and stewardship programs.

#### Epigenomics and regulatory RNAs in AMR

**Figure 8:**
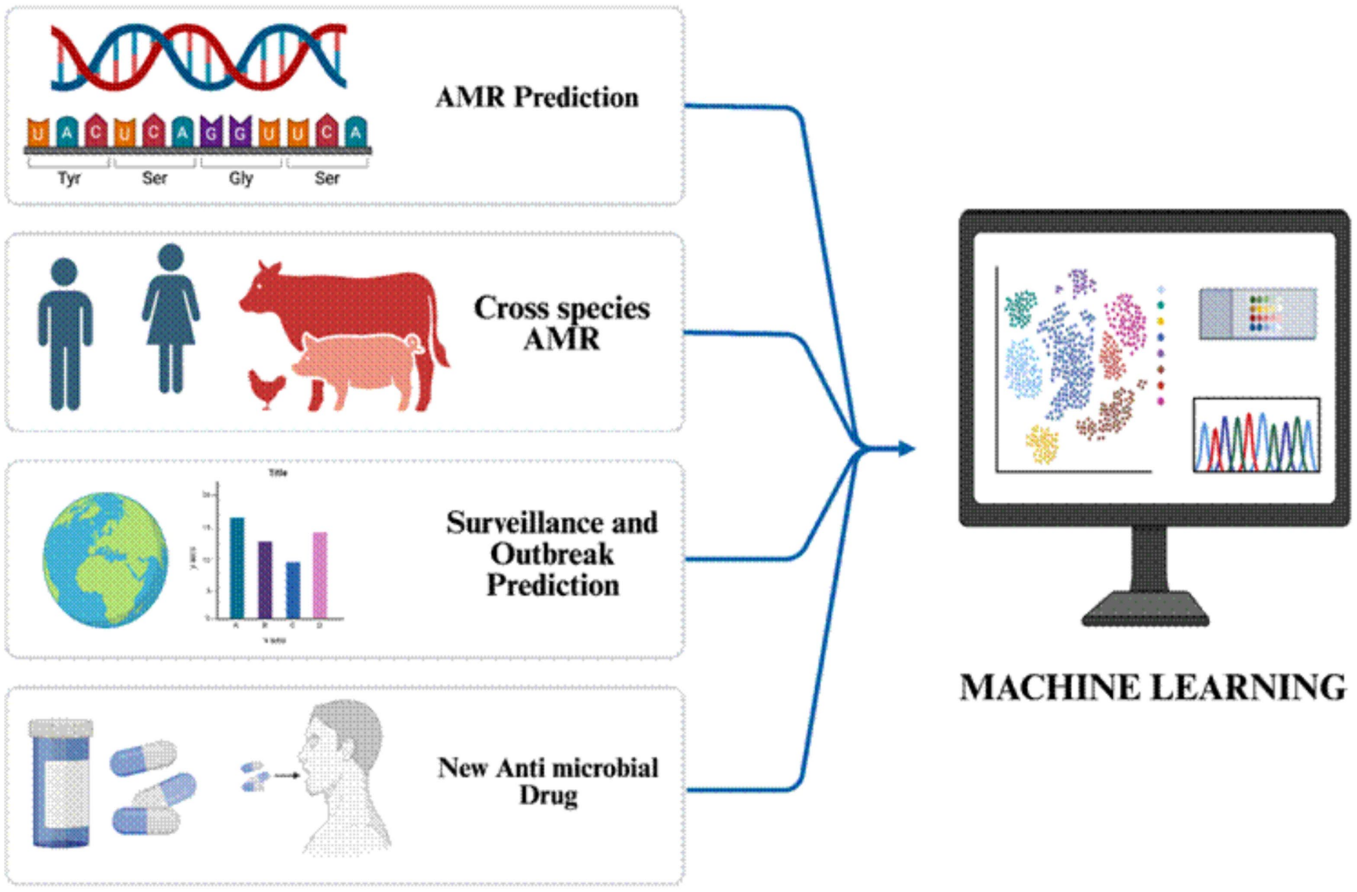
Applications of machine learning in combating antimicrobial resistance (AMR).

Epigenomic and regulatory RNA plays central roles in DEC resistance behaviour. DNA methylation systems, involving Dam and Dcm dependent methyltransferases and Type I restriction modification modules, regulate membrane permeability, porin switching, efflux expression, stress tolerance and persister formation enabling adaptive antibiotic survival without changing ARG content (D’Aquila et al., 2023; Mehershahi and Chen, 2021). Epitranscriptomic modifications, especially rRNA/tRNA methylation and chemical editing modifies ribosomal performance and translation efficiency under drug stress, showing a rapid-response tolerance mechanism that increases genomic resistance traits (Mehershahi and Chen, 2021; Milenkovic and Novoa, 2025). Small regulatory RNAs such as MicF, MicC, RyhB, ArcZ and envelope-stress RNAs dynamically adjust cell envelope composition, oxidative defense pathways, metabolism and motility, arranging multilayered antibiotic tolerance circuits (Yang et al., 2025). These regulatory systems create genotype–phenotype mismatches where isolates with identical ARG repertoires show different susceptibility profiles, complicating resistance prediction and clinical decision making. Targeting RNA mediated circuits, methylation dependent efflux tuning, and biofilm-related tolerance pathways is appearing as a precision therapy strategy, aiming to resensitize resistant DEC populations and disrupt chronic intestinal colonization (see Figure 8).

### One health and controlling global AMR: a new strategic lens

Managing antimicrobial resistance (AMR) in diarrheagenic *Escherichia coli* (DEC) is a complex issue that lies at the intersection of human health, animal agriculture, environmental management, and global interconnection (Gilligan, 1999; Liu et al., 2022; Heredia and García, 2018). Growing research indicates that rather than treating these overlapping systems independently, long-term solutions must recognize and address them. Diarrheagenic *E. coli* (DEC) isolated from different host species show a significant degree of genetic relatedness, according to recent studies. Strains recovered from livestock, poultry, and wild animals often share virulence characteristics and antimicrobial resistance genes with human isolates (Gilligan, 1999; Liu et al., 2022; Heredia and García, 2018; Ren et al., 2024). Ongoing global surveillance of meat and produce consistently detects multidrug-resistant DEC, emphasizing the ease with which cross-contamination and transmission can occur (Ren et al., 2024; Heredia and García, 2018). The spread of diarrheagenic *E. coli* (DEC) is greatly aided by water. Surface waters and irrigation channels are frequently reported to contain DEC in places with poor sanitation or insufficient wastewater treatment, raising dangers for people and animals downstream (Gilligan, 1999; Odumosu et al., 2013; Xiong et al., 2024). The establishment and maintenance of resistance bacteria are further encouraged by the presence of antibiotic residues in these settings. Furthermore, severe weather, especially floods, makes it easier for these infections to spread, highlighting how climatic variability affects the dynamics of microbial transmission(Gilligan, 1999; Odumosu et al., 2013; Xiong et al., 2024). The global expansion of multidrug-resistant DEC is mostly attributed to human activity. Multidrug-resistant DEC is frequently brought home by travelers from high-burden areas, and infection can move great distances due to international food trade (Treacy et al., 2019). Integrated, real-time surveillance that connects environmental, animal, and human health systems is necessary to combat this growing threat. Veterinary, environmental, and public health laboratories must work closely together to effectively monitor resistant clones, with the help of molecular surveillance techniques. Actions will remain reactive rather than preventive in the absence of such integrated systems.

Consistent antimicrobial stewardship in the agriculture and medical sectors is also necessary for effective progress. Increasing the use of quick diagnostic techniques for diarrheal sickness, limiting the use of non-therapeutic antibiotics in livestock, and improving farm and slaughterhouse cleanliness procedures have all demonstrated promising outcomes in various contexts. However, these measures need to be adopted more widely and enforced uniformly (Cantón and Coque, 2006; Odumosu et al., 2013; Prestinaci et al., 2015; Laxminarayan et al., 2016). Importantly, low- and middle-income countries, which face the greatest disease burden, cannot shoulder this responsibility alone. Maintaining international AMR targets requires sustained global investment in laboratory infrastructure, diagnostic instruments, vaccines, and surveillance networks (Khalil et al., 2021; Guarino et al., 2015). In the end, a successful One Health approach functions as a single system rather than a collection of disparate projects. It entails overcoming geographical and disciplinary barriers, integrating surveillance and stewardship across the whole pathway from agriculture to clinical care and making sure that each country takes part in determining global AMR priorities.

## Conclusion

Diarrheagenic *Escherichia coli* (DEC) remains a leading contributor to global diarrhoeal illness and deaths, with the heaviest toll on children in low- and middle-income countries. Evidence gathered over the past decade (2015–2025) points to important changes in its epidemiology: EAEC is emerging as a dominant pathotype in several endemic regions, atypical EPEC and a wider range of non-O157 STEC serogroups are being detected more frequently, and zoonotic as well as foodborne transmission routes are gaining stronger recognition. Antimicrobial resistance is now firmly established across all major DEC types. Particularly troubling are the upward trends in resistance to azithromycin, fluoroquinolones, and third-generation cephalosporins. The interaction of multiple resistance mechanisms such as ESBL production, overactive efflux pumps, reduced outer-membrane permeability, and biofilm-related tolerance has produced highly resilient multidrug-resistant strains that significantly limit the effectiveness of standard treatments. A range of promising therapies such as bacteriophages, targeted vaccines, anti-virulence agents, and microbiota-focused treatments are being explored, yet it is clear that none of these solutions alone can resolve the problem. A comprehensive approach that incorporates One Health surveillance, robust antimicrobial stewardship, improved sanitation systems, and increased funding for quick diagnostics and preventive actions is necessary for real progress. Ultimately, curbing resistant DEC, maintaining antimicrobial efficacy, and reducing the global burden of diarrheal illness will require a coordinated global approach backed by trustworthy local data, sustained international cooperation, and equitable access to therapies.
